# Adenovirus prime, Env protein boost vaccine protects against neutralization-resistant SIVsmE660 variants in rhesus monkeys

**DOI:** 10.1038/ncomms15740

**Published:** 2017-06-05

**Authors:** Brandon F. Keele, Wenjun Li, Erica N. Borducchi, Joseph P. Nkolola, Peter Abbink, Bing Chen, Michael S. Seaman, Dan H. Barouch

**Affiliations:** 1 AIDS and Cancer Virus Program, Leidos Biomedical Research Inc., Frederick National Laboratory for Cancer Research, Frederick, Maryland 21702, USA; 2 Division of Preventive and Behavioral Medicine, University of Massachusetts Medical School, Worcester, Massachusetts 01655, USA; 3 Center for Virology and Vaccine Research, Beth Israel Deaconess Medical Center, Boston, Massachusetts 02215, USA; 4 Children's Hospital, Boston, Massachusetts 02115, USA; 5 Ragon Institute of MGH, MIT and Harvard, Boston, Massachusetts 02139, USA

## Abstract

Previous studies have shown that DNA prime, Ad5 boost vaccines protect against neutralization-sensitive but not neutralization-resistant virus variants within the SIVsmE660 swarm. Here we show that Ad prime, Env protein boost vaccines protect against neutralization-resistant SIVsmE660 variants. We perform two studies in rhesus monkeys with Ad35/Ad26 vectors expressing SIVmac239 Gag/Pol/Env with or without an AS01_B_-adjuvanted SIVmac32H gp140 protein boost. In a repetitive, low-dose challenge study, we observe robust protection against acquisition of infection by both Ad Alone and Ad/Env vaccines. In a single, high-dose challenge study, only the Ad/Env vaccine affords significant protection against acquisition of infection. Analysis of transmitted/founder (T/F) viruses from this study demonstrates that the Ad/Env vaccine blocks both neutralization-sensitive and neutralization-resistant SIVsmE660 variants in rhesus monkeys with restrictive TRIM5α alleles. These data demonstrate that the adjuvanted Env protein boost is critical for protecting against high-dose SIVsmE660 challenge and for blocking neutralization-resistant viruses within the SIVsmE660 swarm.

## Abstract (web summary)

Protection from neutralization-resistant SIV variants is particularly difficult to achieve by vaccination. Here, Keele *et al*. use sieve analysis and show that TRIM5a restrictive rhesus monkeys are protected from neutralization-resistant SIVsmE660 variants by an adenovirus prime, env protein boost vaccine.

The goal of an effective AIDS vaccine is to prevent acquisition of infection following HIV-1 exposure. Most primary HIV-1 isolates have a neutralization-resistant phenotype. Previous studies have reported that a DNA/Ad5 vaccine partially protected against SIVsmE660 challenges but not against SIVmac251 challenges in rhesus monkeys^1^. Following the lack of efficacy of the DNA/Ad5 vaccine in humans^2^, the SIVsmE660 challenge model has generally been viewed as insufficiently stringent for preclinical testing of AIDS vaccine candidates. However, a follow-up study revealed that the DNA/Ad5 vaccine only protected against highly neutralization-sensitive viral variants within the SIVsmE660 swarm and did not protect against neutralization-resistant viral variants within the challenge stock^3^. A particular viral genotype (A/K at position 45/47 of Env) was associated with breakthrough infections, and this ‘A/K genotype' was associated with antibody neutralization resistance^3^,^4^.

We have previously reported that Ad35/Ad26 vectors expressing Gag/Pol/Env provided significant protection against acquisition of neutralization-resistant SIVmac251 challenges but only when Env was included in the adenovirus vaccine^5^. We further showed that addition of an AS01_B_-adjuvanted Env gp140 protein boost augmented protection against neutralization-resistant SIVmac251 challenges^6^.

Several simian immunodeficiency virus (SIV) stocks exist for use in rhesus monkeys, each with benefits and limitations. The two most common lineages used are the molecular clone SIVmac239 with its related isolate SIVmac251, and the molecular clone SIVsmE543 and its related isolate SIVsmE660. These two lineages differ by ∼20% in Env. Importantly, the SIVsm lineage consists of a heterogeneous population consisting of both neutralization-sensitive (T/R at position 45/47) and neutralization-resistant (A/K at position 45/47) viruses. In contrast, the SIVmac lineage is composed primarily of neutralization-resistant (A/K) viruses^7^,^8^,^9^.

A single, high-dose (SHD) intrarectal challenge model is a stringent model to evaluate protective efficacy and provides a robust and consistent infection that is characterized by many more transmitted/founder (T/F) variants establishing systemic infection than are observed in most human infections^10^,^11^,^12^. A repetitive, low-dose (RLD) challenge model is a less stringent model that results in fewer T/F variants establishing systemic infection and can be used to calculate a per-exposure reduction in acquisition risk as one measure of vaccine efficacy^3^,^5^. Combining acquisition data with an enumeration of T/F variants provides further insight into vaccine efficacy^3^. Whether SHD or RLD challenge models are more predictive of clinical efficacy of a vaccine, however, remains to be defined.

Identifying a viral population that can be blocked by vaccination compared to those which can still initiate infection is a powerful tool to identify potential mechanisms of protection^13^. Genetic sieve analyses are designed to identify viral lineages that are capable of initiating infection but have been blocked by vaccine-induced immune responses. In human vaccine trials, sieve analyses are limited due to the enormous heterogeneity of HIV-1 in a population such that each patient is infected with a unique viral genome. For these analyses, a comparison is made of the overall genetic distance of each breakthrough infection to the vaccine strain, as well as observing single amino acid polymorphisms that differ between vaccinees and placebo controls. If these polymorphisms deviate from the vaccine strain, it is considered vaccine-induced selection^13^. In rhesus monkey challenge studies, the benefits of sieve analyses are expanded since the genetic makeup of the inoculating virus itself is known and all animals are infected with the same inoculum, providing an important control in establishing which viral lineages initiate infection in both vaccinated and control animals^3^.

Here we show that Ad35/Ad26 vectors expressing Gag/Pol/Env followed by an adjuvanted Env gp140 protein boost protected against both neutralization-sensitive and neutralization-resistant virus variants within the SIVsmE660 swarm. We performed parallel studies using both SHD and RLD SIVsmE660 challenge models to compare protective efficacy and the number of T/F variants, and we demonstrate that this vaccine could protect against A/K neutralization-resistant viral variants within the SIVsmE660 swarm by a sieve analysis.

## Results

### Repetitive low-dose challenge study

**Figure 1:**
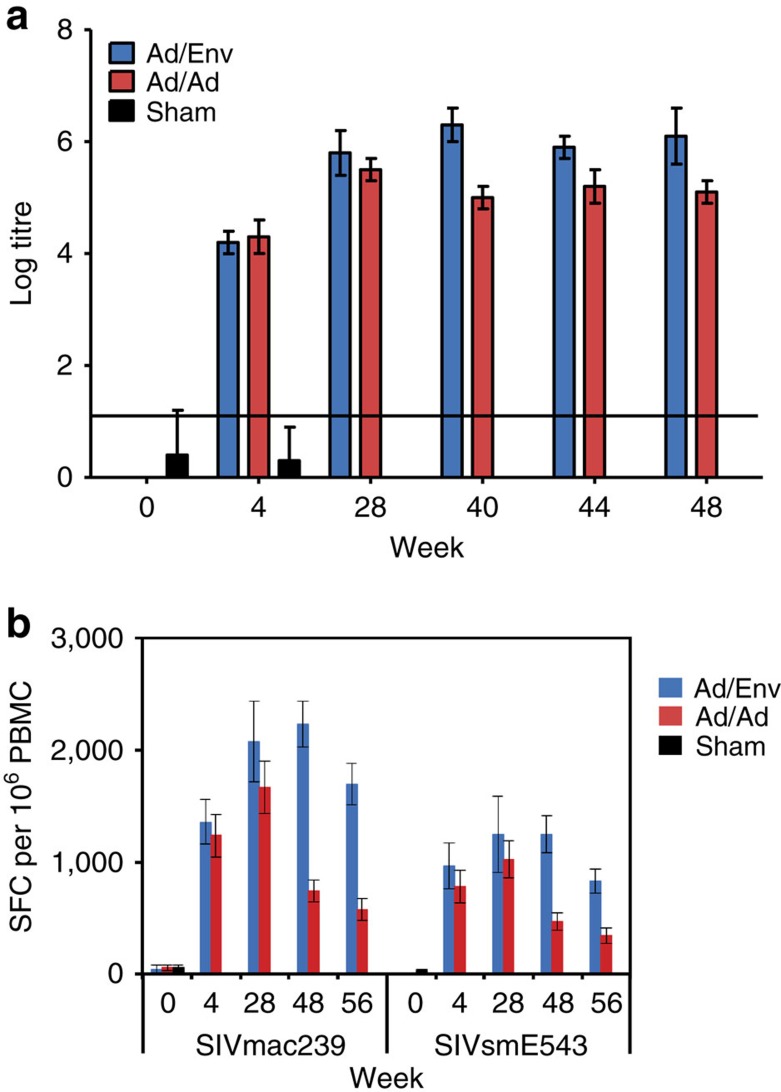
Humoral and cellular immune responses. Vaccine-induced binding antibodies titres (*N*=12/group) were determined by SIVmac251 Env ELISA at week 0, 4, 28, 40, 44 and 48 following vaccination (**a**). Cellular immune responses to SIVmac239 and SIVsmE543 Gag, Pol and Env were determined by IFN-γ ELISPOT assays at weeks 0, 4, 28, 48 and 56 and reported as spot-forming cells (SFC) per million PBMC (**b**). Ad/Env (blue) indicates animals primed with Ad35/Ad26 and boosted with adjuvanted Env gp140 (Ad35/Ad26/Env). Ad/Ad (red) indicates animals vaccinated with Ad35/Ad26 only. Sham controls (black) were vaccinated with Ad35/Ad26 expressing no relevant antigens. Error bars represent s.e.m.

In Study 1 (RLD challenge study), 36 animals were grouped into three arms (*N*=12 per group): Ad35/Ad26 only, Ad35/Ad26/Env or sham controls. Vaccinated animals received 2 × 10^10^ viral particles Ad35 (ref. 14) expressing SIVmac239 Gag/Pol/Env at week 0 and 2 × 10^10^ viral particles Ad26 expressing the same SIVmac239 Gag/Pol/Env inserts at week 24. In the Ad35/Ad26/Env group, animals were boosted with 0.25 mg SIVmac32H Env gp140 with GSK Adjuvant System AS01_B_ at weeks 36, 40 and 44. Binding antibody titres were measured at weeks 0, 4, 28, 40, 44 and 48 (Fig. 1a; Supplementary Table 1). Ad35/Ad26 and Ad35/Ad26/Env vaccinated animals had antibody titres from week 4 onwards. Following the Env protein boost, the Ad35/Ad26/Env vaccinated animals developed ∼1 log higher titres of binding antibodies than the Ad35/Ad26 vaccinated animals. Cellular immune responses were measured by IFN-γ ELISPOT assays at weeks 0, 4, 28, 48 and 56 against homologous peptides and heterologous peptides (Fig. 1b; Supplementary Table 1). Responses to the heterologous SIVsmE543 peptides were ∼2-fold lower than responses to the homologous SIVmac239 peptides. Following the Env protein boost, the Ad35/Ad26/Env group had higher ELISPOT responses than Ad35/Ad26. The immunogenicity of this vaccine essentially was comparable with our previously published studies^6^.

**Figure 2:**
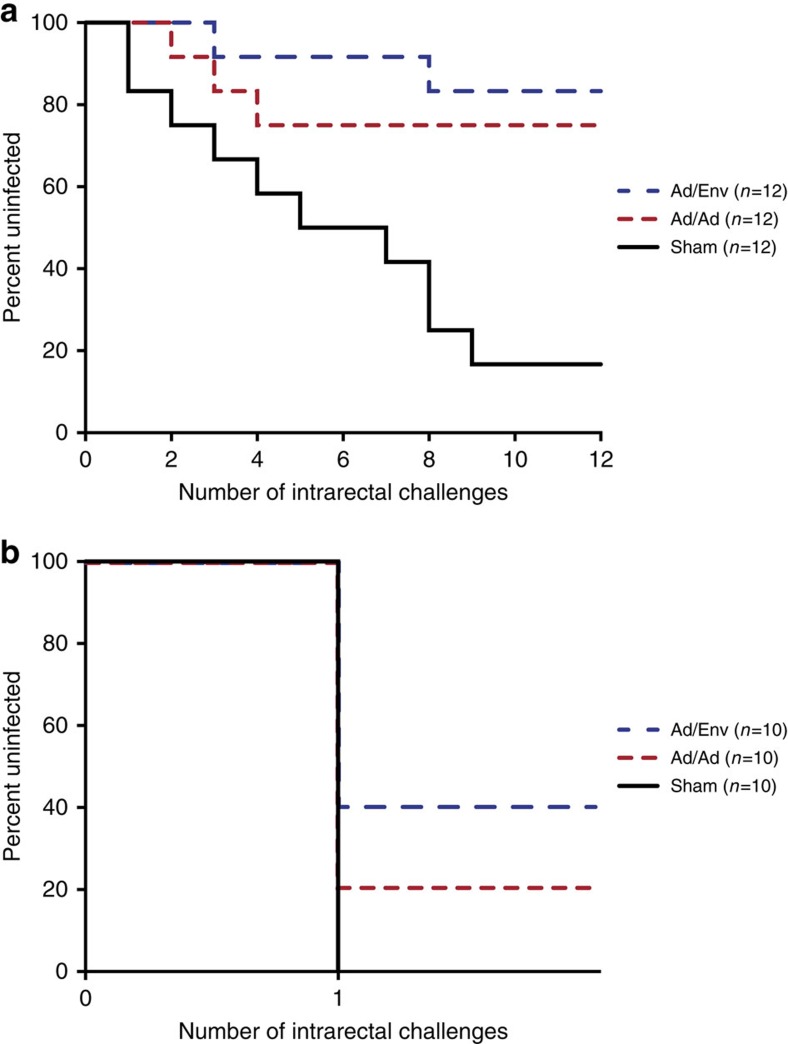
Protective efficacy. The number of challenges required for detection of productive infection for each vaccine group. Kaplan–Meier plot indicates the percent of uninfected animals after each challenge. Ad/Env (blue) indicates animals primed with Ad35/Ad26 and boosted with adjuvanted Env gp140 (Ad35/Ad26/Env). Ad/Ad (red) indicates animals vaccinated with Ad35/Ad26 only. The RLD challenge study (**a**) shows infections over time with protection in 75% of Ad/Ad vaccinated animals and 85% of Ad/Env vaccinated animals. The SHD challenge study (**b**) shows infections in all control animals and protection in 20% of Ad/Ad vaccinated animals and in 40% protection in Ad/Env vaccinated animals.

Animals were challenged weekly for 12 weeks by a repetitive, low-dose (RLD) challenge protocol with a 1:1,000 dilution (930 TCID_50_) of our SIVsmE660 stock by the intrarectal route. Following 12 challenges, 10 of 12 sham vaccinated animals were infected, as compared with 3 of 12 Ad35/Ad26 vaccinees and 2 of 12 Ad35/Ad26/Env vaccinees (Fig. 2a). Both vaccinated groups demonstrated statistically significant protection from acquisition of infection (Table 1). The Ad35/Ad26 group showed 75% complete protection with a Cox proportional hazard ratio of 0.194 (0.051–0.072 95% CI, *P*=0.016) and a per-exposure risk reduction of 80.6%, consistent with prior studies^5^. The Ad35/Ad26/Env group demonstrated 83% complete protection with a Cox proportional hazard ratio of 0.114 (0.024–0.541 95% CI, *P*=0.006) and a per-exposure risk reduction of 88.6%. Therefore, in the RLD challenge model, both vaccinated arms showed robust protection against acquisition of infection.

**Figure 3:**
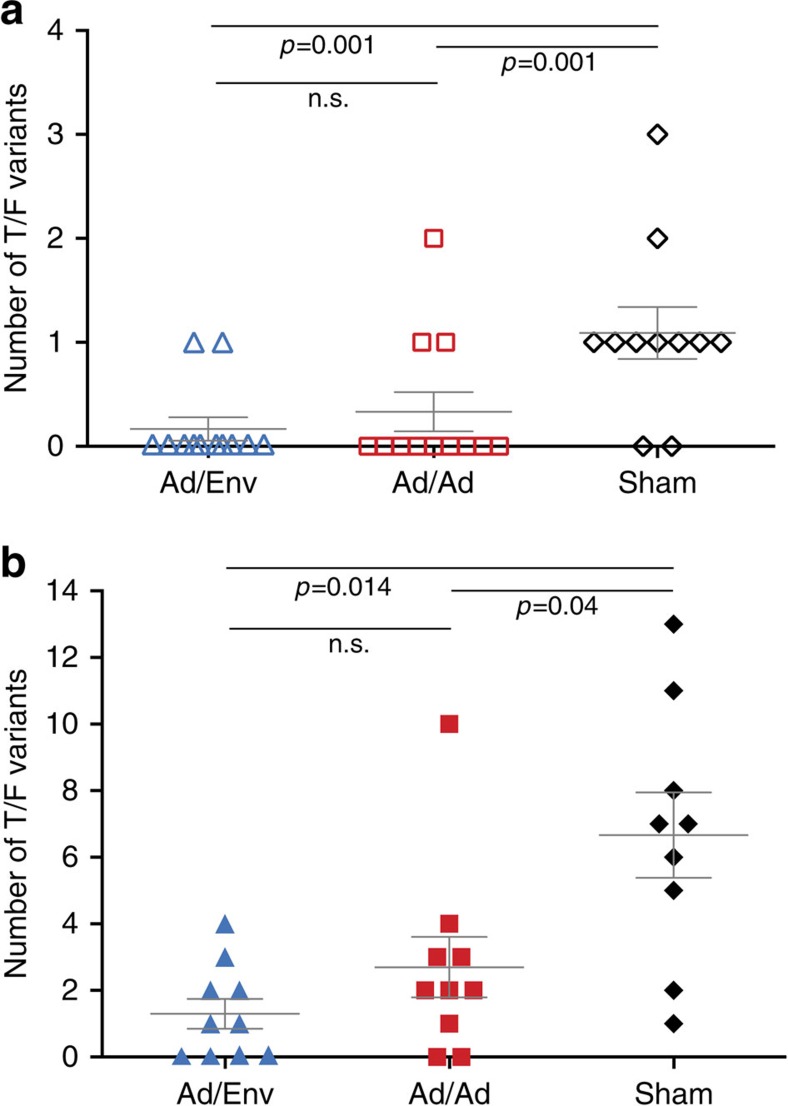
Transmitted/founder analysis. The total number of T/F genomes was determined in each animal in the RLD (**a**) and SHD (**b**) challenge studies. Average and standard error are indicated. Significant differences in mean number of variants by Wilcoxon rank-sum tests are shown. Ad/Env (blue) indicates animals primed with Ad35/Ad26 and boosted with adjuvanted Env gp140 (Ad35/Ad26/Env). Ad/Ad (red) indicates animals vaccinated with Ad35/Ad26 only. Difference between vaccinated groups were not significant (n.s.).

Comparing the number of T/F variants between vaccine arms is a direct measure of vaccine efficacy and can be integrated with acquisition data to provide a more comprehensive view of vaccine protection^3^. We therefore performed single genome amplification (SGA) and sequence analysis to determine the number of transmitted/founder (T/F) viruses in each animal. We obtained 319 full-length *env* sequences and inferred the number of T/F genomes by phylogenetically comparing all sequences from infected animals as well as from the original virus inoculum (Supplementary Fig. 1A). This composite tree was used as a guide to distinguish closely related viruses and identify independent infection events. All sequences from each animal were also analysed individually using phylogenetic approaches and visual inspection of alignments (Supplementary Fig. 1B–D), as well as mathematical models of early viral diversification^10^,^11^,^15^,^16^. Using this approach, individual lineages representing unique T/F genomes that established systemic infection were inferred. The number of T/F variants detected in the plasma of all animals ranged from a single variant to 3 identifiable variants (Fig. 3a). The median number of variants initiating infection in sham controls was 1 (mean of 1.2; range 0–3), which was significantly reduced in the Ad35/Ad26 arm, which had a median number of variants of 0 (mean 0.33; range 0–2; *P*=0.001, Wilcoxon rank-sum test) and was further reduced in the Ad35/Ad26/Env arm with a median number of variants of 0 (mean 0.17; range 0–1; *P*=0.001, Wilcoxon rank-sum test). There is no significant difference between vaccinated groups (*P*=0.423, Wilcoxon rank-sum test). The relatively low number of T/F variants in all groups is consistent with the RLD challenge model and highlights the uniformity of infection utilizing this approach.

We next sought to assess the differences in protective effects of each vaccine stratified by their neutralization genotype: neutralization resistant A/K or neutralization sensitive non-A/K (Supplementary Fig. 2; Table 2). For the ten of twelve infected control animals, eight were neutralization sensitive (non-A/K) and two were neutralization resistant (A/K). For the three of twelve infected Ad/Ad animals, two were neutralization sensitive (non-A/K) and one was neutralization resistant (A/K). For the two of twelve infected Ad35/Ad26/Env (Ad/Prot) animals, both were neutralization resistant (A/K). We found a significant protective effect in both vaccine arms when comparing total infection events and non-A/K viruses. However, there was insufficient power to evaluate a potential vaccine effect on A/K viruses as a result of the low number of infections in the vaccinated animals (three and two infection events for Ad35/Ad26 only and Ad35/Ad26/Env, respectively). The results were largely invariant to choice of statistical models and varied statistical assumptions (Table 2). Therefore, while both vaccines in the RLD study showed statistically significant protection and a reduction in the number of T/F viruses, there was insufficient power to assess protection specifically against neutralization-resistant A/K variants due to the high level of protection observed in this study.

### Single high-dose challenge study

We reasoned that a high-dose viral challenge should result in increased infection events that would facilitate the sieve analysis and thus may allow for an assessment of vaccine efficacy specifically against neutralization-resistant A/K SIVsmE660 variants. Therefore, in Study 2 (SHD challenge study), 30 rhesus monkeys were grouped into three arms (*N*=10 per group) and received the same vaccines as in Study 1: Ad35/Ad26 only, Ad35/Ad26/Env or sham controls. Humoral and cellular immune responses were comparable with the prior study (Supplementary Fig. 3; Supplementary Table 1). In this study, all animals had resistant TRIM5α alleles except one animal in each vaccine group. Animals were then challenged by the intrarectal route with a single 9.3 × 10^5^ TCID_50_ dose of undiluted SIVsmE660, which is 1,000-fold higher than the dose used in Study 1 (RLD challenge study). All 10 sham vaccinated animals were infected after this single, high-dose challenge. In contrast, eight of ten Ad35/Ad26 vaccinated animals and only six of ten Ad35/Ad26/Env vaccinated animals became infected (Fig. 2b). Protection against acquisition of infection was significant in the Ad35/Ad26/Env group (40%; *P*=0.043, Fisher's exact test) but not in the Ad35/Ad26 group (20%; *P*=0.237, Fisher's exact test) (Table 1). These data suggest that the Env protein boost was important for optimizing protective efficacy against high-dose challenge with SIVsmE660.

We next identified the number and genetic characteristics of all T/F variants following challenge in this study. After the SHD challenge, the median number of T/F variants was seven in the sham controls (mean 6.7, range 1–13), which was significantly reduced in the Ad35/Ad26 vaccinated animals to a median of only two variants (mean 2.7; range 0–10, *P*=0.04, Poisson regression), and further reduced in the Ad35/Ad26/Env vaccinated animals to a median of 1 variant (mean 1.3; range 0–4, *P*=0.014, Poisson regression) (Fig. 3b). Increasing the total number of T/F variants with the SHD challenge model allowed us to quantitate more clearly the reduction in the number of variants establishing infection in the vaccinated arms. Interestingly, using a fractional polynomial regression model, we identified a strong, non-linear relationship between the number of T/F variants and peak viremia (*P*<0.001, likelihood ratio test).

### Evaluation of breakthrough infections

**Figure 4:**
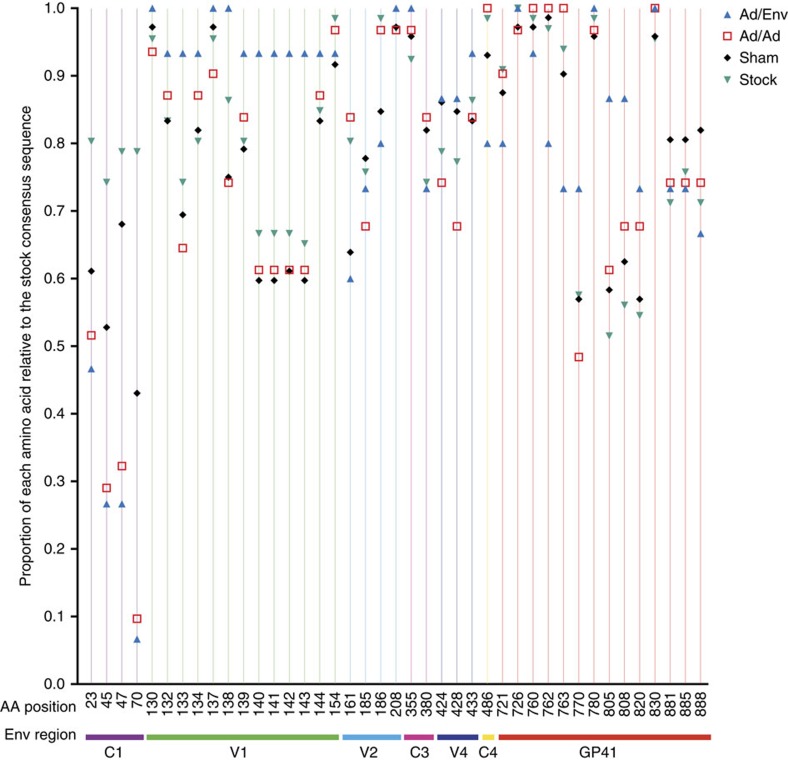
Genetic sieve analysis. The entire Env proteome for each T/F virus and all stock sequences were compared to the consensus sequence of the stock. All 41 informative sites are plotted as the proportion of each amino acid relative to the consensus sequence of the stock. Gene regions are colour coded and labelled. For most sites, the majority of T/F virus sequences match the stock consensus sequence. However, amino acids 45, 47 and 70 showed significant enrichment in vaccinated animals demonstrating the sieve effect for viruses with nonconsensus changes at these sites. Statistical comparisons for all sites are shown in Supplementary Table 2.

We next evaluated breakthrough infections in both the RLD and SHD studies. We identified 118 T/F variants in total (15 from Ad35/Ad26/Env vaccinees, 31 from Ad35/Ad26 vaccinees, and 72 from sham controls). Genetic sieve analysis was performed using the inferred amino acid translation over the entire Env for all 118 T/F genomes, as well as 66 inoculum sequences. Each T/F lineage was compared to the consensus sequence of the stock and plotted as a fraction of this consensus for each vaccine group (Fig. 4). Fisher's Exact Permutation Test was then used to identify the sites with significant differences between vaccinated and sham controls. In total, 41 sites were identified as informative with more than one T/F genome containing polymorphisms at each site (Supplementary Table 2). The vast majority of all 41 polymorphisms were apparent in both vaccinated and control animals indicative of random variation without vaccine selection. However, there were a few sites statistically enriched only in vaccinated animals. These include three single amino acid mutations (T45A, R47K, S70N) that were statistically enriched in both Ad35/Ad26 and Ad35/Ad26/Env vaccinated animals. The asparagine at site 70 produces a potential N-linked glycosylation (PNG) site and is found in only 21% of the stock sequences. This minor population was significantly enriched in breakthrough viruses representing 90% of T/F viruses in the Ad35/Ad26 group (*P*=0.001 compared to sham, Fisher's exact test) and 93% of T/F in the Ad35/Ad26/Env arm (*P*=0.008 compared to sham, Fisher's exact test). The neutralization resistant A/K genotype (T45A, R47K) was also highly enriched in breakthrough viruses in vaccinated animals. We found the A/K genotype in 68% of the T/F viruses in the Ad35/Ad26 group (*P*=0.001 compared with sham, Fisher's exact test) and in 73% of the T/F in the Ad35/Ad26/Env group (*P*=0.007 compared to sham, Fisher's exact test). In addition to these single amino acid polymorphisms, length variation within the V1 loop was also associated with breakthrough infections in Ad35/Ad26 vaccinated animals (*P*=0.015 compared to sham, Fisher's exact test) and Ad35/Ad26/Env vaccinated animals (*P*=0.035 compared with sham, Fisher's exact test). Therefore, genetic sieve analyses of T/F viruses from vaccinated and control animals revealed selection of viruses in vaccinated animals with a critical N-linked glycan, an increase in proportion of neutralization-resistant A/K viruses, and mutations within variable loops. Taken together, these data provide evidence for vaccine-elicited protection with the greatest selection seen in the Ad35/Ad26/Env vaccinated animals.

We next sought to assess the differences in protective effects of each vaccine stratified by their neutralization genotype: neutralization resistant A/K or neutralization sensitive non-A/K. This analysis could only be performed in the SHD study as a result of the increased number of infection events. Protective effects against neutralization-resistant A/K viruses were expressed, as incidence rate ratios (IRR) and assessed using a Poisson regression model (Table 3). As expected, analysis of the neutralization-sensitive non-A/K variants demonstrated significantly lower IRRs compared with sham controls in both Ad35/Ad26 (*P*<0.001, Poisson regression) and Ad35/Ad26/Env (*P*<0.001, Poisson regression) vaccinated animals. Remarkably, in the neutralization-resistant A/K analysis, we also observed a significant reduction in the IRR compared with sham controls in the Ad35/Ad26/Env vaccinated animals (*P*=0.050, Poisson regression), but not in the Ad35/Ad26 vaccinated animals (*P*=0.876, Poisson regression). The two animals with a permissive TRIM5α genotype were protected and thus did not impact this sieve analysis. These data demonstrate that the Env gp140 protein boost was required to block the A/K neutralization-resistant variants within the SIVsmE660 swarm.

**Figure 5:**
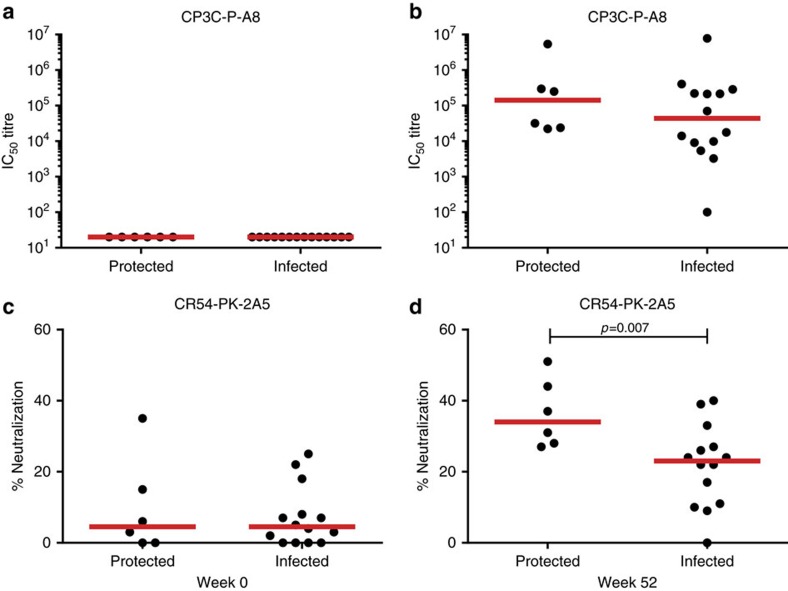
Correlation of protective efficacy with neutralizing antibody responses. Neutralizing antibody responses are shown at week 0 (**a**,**c**) and week 52 (**b**,**d**) in the vaccinated animals against both the non-AK neutralization-sensitive tier 1 SIVsmE660 clone (CP3C-P-A8) and the AK neutralization-resistant tier 2 SIVsmE660 clone (CR54-PK-2A5). IC_50_ titres are reported for CP3C-P-A8. % neutralization values are reported for CR54-PK-2A5. Wilcoxon rank-sum tests were utilized to compare neutralizing antibody titres in protected versus infected vaccinated animals at week 52. Red line indicates median responses.

We evaluated neutralizing antibody activity in the vaccinated animals against both the non-AK neutralization-sensitive tier 1 SIVsmE660 clone (CP3C-P-A8) and the AK neutralization-resistant tier 2 SIVsmE660 clone (CR54-PK-2A5). The vaccine induced high tier 1 NAb titres and low but detectable tier 2 NAb responses, which are reported as % neutralization. As shown in Fig. 5, the protected animals induced higher tier 2 NAb responses to the AK neutralization-resistant variant CR54-PK-2A5, as compared with the infected animals at week 52 (*P*=0.007, Wilcoxon rank-sum test), which is consistent with the sieve analysis. Taken together, these data demonstrate that the Ad35/Ad26/Env vaccine afforded 40% protection against acquisition of a heterologous, single, high-dose challenge with SIVsmE660 and blocked not only neutralization-sensitive non-A/K viruses but also neutralization-resistant A/K viruses within the swarm.

## Discussion

We recently reported that an Ad26-Gag/Pol/Env prime, Env gp140 boost vaccine afforded 50% efficacy against heterologous RLD challenges with neutralization-resistant SIVmac251 (ref. 6). In the present study, we confirmed and extended these findings using an Ad-Gag/Pol/Env prime, Env gp140 boost vaccine against heterogeneous RLD and SHD challenges with SIVsmE660. The Ad prime, Env protein boost vaccine significantly protected 40% of animals against the high-dose SIVsmE660 challenge. Using a sieve analysis of the T/F viruses, we demonstrated that this vaccine protected not only against neutralization-sensitive non-A/K viruses but also against neutralization-resistant A/K viruses. To the best of our knowledge, this is the first report of protection against neutralization-resistant A/K variants within the SIVsmE660 swarm by any vaccine. These data highlight the protective efficacy of the Ad prime, Env protein boost vaccine and suggest the importance of the Env protein boost in blocking neutralization-resistant viral variants.

Protection against RLD SIVsmE660 challenges has also been reported for a DNA prime, Ad5 boost vaccine^3^. In this prior study, no protection was observed against neutralization-resistant A/K variants within the SIVsmE660 swarm. In the present study, we observed protection against both RLD and SHD SIVsmE660 challenges, and the sieve analysis revealed that there were only a limited number of sites under selection pressure following transmission in vaccinated animals. These included the A/K sites at positions 45/47, which were initially postulated^3^ and subsequently proven^4^ to confer a neutralization-resistant phenotype. We also found evidence of selection of an N-linked glycan at position 70 that was significantly enriched in vaccinated animals. Altogether, this A-K-N genotype in breakthrough viruses suggests that vaccine efficacy was antibody mediated.

We previously reported significant differences between diverse SIVmac251 stocks^17^. In contrast, SIVsmE660 is generally more homogeneous with most stocks containing overlapping viral genomes. One exception is a prior study in which an SIVsmE660 stock had an unusually low frequency of neutralization-resistant A/K viruses^18^. In that study, there was no evidence of selection for A/K viruses with a DNA prime, MVA boost vaccine likely due to the limited proportion of A/K variants in the stock (5%). While these authors found an enrichment in all transmission events for A/K viruses, there was no increase in selection in vaccinated animals. It was suggested that all founder viruses are enriched for the A/K genotype^18^,^19^. While we also found an enrichment in the A/K genotype compared to the stock, the vaccine effect we report here was a statistically significant increase in the A/K genotype in vaccinated animals compared to sham controls. Therefore, while A/K viruses are likely found more often in all infection events, its further enrichment is indicative of a vaccine protective effect. Importantly, when the A/K genotype was first reported in a DNA prime, Ad5 boost immunization study^3^, Roederer *et al*. found that 23.5% of that stock contained the resistant A/K form of the virus. In the SIVsmE660 stock reported here, 21% of viruses contained the resistant A/K genotype, which allowed for both a meaningful sieve analysis and direct comparisons between studies. Therefore, unlike the DNA prime, Ad5 boost study^3^, we found significant protection against neutralization-resistant A/K viruses in the SIVsmE660 swarm using comparable virus stocks. These data are consistent with our previous findings with SIVmac251, in which most of the viruses in the SIVmac251 stock are neutralization-resistant^6^.

The ability to protect against neutralization-resistant viruses within a swarm is important for a candidate HIV-1 vaccine, since many primary isolate viruses contain neutralization-resistant viruses. Moreover, our findings show the utility of genetic sieve analyses in analysing vaccine efficacy to gain a greater level of understanding beyond the overall protective efficacy of the vaccine. Together with our previous study^6^, the Ad prime, Env protein boost strategy has shown protection in multiple challenge models (RLD SIVmac251, RLD SHIV-SF162P3, RLD SIVsmE660, SHD SIVsmE660), thus supporting further clinical development of this vaccine concept.

## Methods

### Immunizations and challenges

Indian-origin, outbred, young adult, male and female, specific pathogen-free (SPF) rhesus monkeys (*Macaca mulatta*) that did not express the controlling class I alleles Mamu-A*01, Mamu-B*08 and Mamu-B*17 were housed at the New England Primate Research Center (NEPRC), Southborough, Massachusetts, USA. A total of 66 animals were used for both studies. Groups were balanced for susceptible and resistant TRIM5α alleles^1^,^20^. Immunizations were performed by the intramuscular route in the quadriceps muscles with 2 × 10^10^ viral particles of Ad35 or Ad26 vector^14^ expressing SIVmac239 Gag-Pol and Env (gp140). Ad35/Ad26 vaccinated animals were primed with Ad35 at week 0 and boosted with Ad26 at week 24. Ad35/Ad26/Env vaccinated animals were primed with Ad35 at week 0 and boosted with Ad26 at week 24 and then given 0.25 mg of SIVmac32H Env gp140 (ref. 21) with GSK Adjuvant System AS01_B_ at weeks 36, 40 and 44. To evaluate protective efficacy and immunological correlates, repetitive, low-dose (RLD) challenges were performed beginning at week 52 with 12 intrarectal inoculations of the heterologous virus SIVsmE660 (ref. 1) using a 1:1,000 dilution (930 TCID_50_) of our challenge stock. Monkeys were bled weekly for viral loads (Siemans Diagnostics), and the date of infection was defined as the last challenge time point before the first positive SIV RNA level. SHD challenges were performed with a 9.3 × 10^5^ TCID_50_ dose of undiluted virus administered intrarectally. In the SHD challenge study, all animals had resistant TRIM5α alleles except one animal in each vaccine group. Animals were followed to determine set point viral loads. All animal studies were approved by the Harvard Medical School Institutional Animal Care and Use Committee (IACUC).

### Cellular and humoral immune assays

SIV-specific cellular immune responses were assessed by IFN-γ ELISPOT assays essentially, as described in ref. 22. ELISPOT assays used pools of SIVsmE543 and SIVmac239 Gag, Pol and Env peptides. Peptides were 15 amino acids in length and overlapped by 11 amino acids. Spot-forming cells (SFC) in 200,000 peripheral blood mononuclear cells (PBMC) were assessed separately in response to Gag, Pol and Env peptide pools. Total responses were defined as the sum of Gag, Pol, and Env responses and were normalized to SFC per million PBMC. SIV-specific humoral immune responses were assessed by SIVmac251 Env ELISA and TZM-bl pseudovirus neutralization assays essentially, as described in refs 1, 23.

### Single genome amplification and T/F enumeration

From each plasma specimen and the challenge inoculum, viral RNA was extracted using the QIAamp Viral RNA Mini kit (Qiagen). RNA was eluted and immediately subjected to cDNA synthesis. Reverse transcription of RNA to single-stranded cDNA was performed using 1 × RT buffer, 0.5 mM of each deoxynucleoside triphosphate, 5 mM dithiothreitol, 2 U ml^−1^ RNaseOUT (RNase inhibitor), 10 U ml^−1^ of SuperScript III reverse transcription and 0.25 mM antisense primer SIVsmEnvR1 5′-TGT AAT AAA TCC CTT CCA GTC CCC CC-3′ (nt 9454–9479 in SIVmac239). The mixture was incubated at 50 °C for 60 min, followed by an increase in temperature to 55 °C for an additional 60 min. The reaction was then heat-inactivated at 70 °C for 15 min and treated with 2 U of RNAse H at 37 °C for 20 min. The newly synthesized cDNA was used immediately or frozen at −80 °C. cDNA was serially diluted and distributed among 96-well plates so as to identify a dilution where PCR-positive wells constituted <30% of the total number of reactions. PCR amplification was performed in the presence of 1 × High Fidelity Platinum PCR buffer, 2 mM MgSO_4_, 0.2 mM of each deoxynucleoside triphosphate, 0.2 μM of each primer and 0.025 U μl^−1^ Platinum Taq High Fidelity polymerase in a 20-μl reaction (Invitrogen). First round PCR primers included sense primer SIVsmEnvF1 5′-CCT CCC CCT CCA GGA CTA GC-3′ (nt 6127–6146 in SIVmac239) and antisense primer SIVsmEnvR1. PCR was performed with the following parameters: 1 cycle of 94 °C for 2 min, 35 cycles of a denaturing step of 94 °C for 15 s, an annealing step of 55 °C for 30 s, and an extension step of 68 °C for 4 min, followed by a final extension of 68 °C for 10 min. Next, 1 μl from first-round PCR product was added to a second-round PCR reaction that included the sense primer SIVsmEnvF2 5′-TAT GAT AGA CAT GGA GAC ACC CTT GAA GGA GC-3′ (nt 6292–6323 in SIVmac239) and antisense primer SIVsmEnvR2 5′-ATG AGA CAT RTC TAT TGC CAA TTT GTA-3′ (nt 9413–9439 in SIVmac239). The second-round PCR reaction was performed under the same conditions used for first-round PCR, but with a total of 45 cycles. All PCR procedures were performed under PCR clean room conditions using procedural safeguards against sample contamination, including prealiquoting of all reagents, use of dedicated equipment, and physical separation of sample processing from pre- and post-PCR amplification steps. Correctly sized amplicons determined by electrophoresis on an agarose gel were directly sequenced by cycle-sequencing using BigDye terminator chemistry and protocols recommended by the manufacturer (Applied Biosystems). Individual sequence fragments for each amplicon were assembled and edited using Sequencher (Gene Codes). Inspection of individual chromatograms allowed for the confirmation that amplicons were derived from a single viral template. The absence of mixed bases at each nucleotide position throughout the entire env gene was taken as evidence of SGA from a single vRNA/cDNA template. This quality control measure allowed us to exclude from the analysis amplicons that resulted from PCR-generated *in vitro* recombination events or Taq polymerase errors and to obtain individual env sequences that proportionately represent those virions circulating *in vivo*.

All sequence alignments and phylogenetic trees were performed in ClustalW with manual editing in MacClade. Each low-diversity lineage was compared with a mathematical model of viral diversification overtime to identify all transmitted/founder lineages, as previously described in ref. 10. The number of TF variants was determined by sequence analysis (phylogenetic trees and highlighter plots) comparing all sequences within an individually infected animal with and without stock sequences. The number of variants identified were confirmed using a phylogenetic tree of all sequences combined. The average number of sequences analysed per animal was 22 (range 16–26) with no significant differences in the number of sequences between groups (*P*=0.4 ANOVA) and no correlation between the number of TF and the number of sequences per animal (*P*=0.73 Pearson).

### Sieve analysis

Each T/F variant from all infected animals (total of 118 T/F lineages), as well as 66 stock sequences were used to generate a consensus amino acid sequence. Each T/F lineage was then compared with the consensus and plotted as a fraction of the consensus for each vaccine group regardless of challenge dose. In total, 41 sites were identified as informative excluding single sequence polymorphisms. Fisher's Exact/Permutation Tests with Bonferroni corrections were utilized to identify significant enrichment of amino acids at each of these 41 sites.

### Statistical analyses

Protection against acquisition of infection was analysed using the Cox proportional hazards model with exact partial-likelihood method to handle tied failures. The number of repeated challenges from inoculation to the earliest detection of SIV infection or censorship was analysed, as a discrete time variable in the Cox regression models. The number of challenges required for 50% infection of each group, hazard ratios with 95% confidence intervals, per-exposure vaccine efficacy and per-exposure risks of infection were quantified. Vaccine efficacy was defined as the reduction in the per-exposure probability of infection as previously described in refs 24, 25. Protection against all T/F, A/K only or non-A/K genomes by experimental group were analysed using log-rank tests, Cox proportional hazards model and exact logistic regression model. For the analysis of the vaccine effects on number T/F, A/K or non-A/K variants per animal, Poisson regression models were used. Uninfected animals were coded as 0 counts of variants^26^,^27^,^28^. Protective effects are expressed as incidence rate ratios (IRR). We then used simultaneous estimation equations to compare the equivalence of protective effects against all T/F, A/K or non-A/K virus. Fisher's exact permutation test was used to compare differences in non-consensus rates between experimental groups. Bonferroni corrections were applied to the eight tests on each site, and a threshold of *P*<0.00625 was therefore used to determine statistical significance.

The association of the peak viral load with number of T/F was analysed using fractional polynomial regression models. A strong non-linear association between the two variables was detected. To properly model the relationship, we transformed the number of T/F value (X) as its inverse (1/X), and used 1/X as the predictor. Wilcoxon rank-sum tests were utilized to compare neutralizing antibody titres in protected versus infected vaccinated animals at week 52.

### Data availability

All 813 sequences from infected animals are deposited in GenBank under accession numbers KP237051–KP237863 and 66 stock sequences KX360059–KX360124. All other relevant data are available from the authors upon request.

## Additional information

**How to cite this article:** Keele, B. F. *et al*. Adenovirus prime, Env protein boost vaccine protects against neutralization-resistant SIVsmE660 variants in rhesus monkeys. *Nat. Commun.* **8,** 15740 doi: 10.1038/ncomms15740 (2017).

**Publisher's note:** Springer Nature remains neutral with regard to jurisdictional claims in published maps and institutional affiliations.

## Supplementary Material

Supplementary Information Supplementary Figures and Supplementary Tables

## Figures and Tables

**Table 1:** Protective efficacy of Ad35/Ad26/Env and Ad35/Ad26 vaccines against RLD and SHD SIVsmE660 challenges.

**Challenge**	**Vaccine**	Complete protection*	***P*** **value versus Sham**	**Hazard ratio (95% confidence interval)**	**Per-exposure risk reduction**
Repetitive low-dose (RLD)	Ad35/Ad26/Env	83%	0.006†	0.114 (0.024–0.541)	88.6%
	Ad35/Ad26	75%	0.016†	0.194 (0.051–0.072)	80.6%
	Sham	16%	N/A	1	N/A
					
Single high-dose (SHD)	Ad35/Ad26/Env	40%	0.043‡	N/A	N/A
	Ad35/Ad26	20%	0.237‡	N/A	N/A
	Sham	0%	N/A	N/A	N/A

^*^Percent uninfected animals after 12 intrarectal challenges.

^†^Cox proportional hazard model.

^‡^Fisher's exact test.

**Table 2:** Cox and exact logistic regression models for protection against all T/F Genomes, A/K-only genomes, or non-A/K genomes in the RLD challenge study.

	**Group**	**No. of animals**	**Log-rank test (*P*-value)**	**Cox model**				**Exact logistic regression model**			
				**Haz. Ratio**	**Lower 95% CI**	**Upper 95% CI**	***P*** **value**	**Odds ratio**	**Lower 95% CI**	**Upper 95% CI**	***P*** **value**
Total Infections	Sham	12	Ref.	Ref.				Ref.			
	Ad35/Ad26	12	**0.0092**	0.194	0.051	0.735	**0.016**	0.077	0.005	0.647	**0.0123**
	Ad35/Ad26/Env	12	**0.0010**	0.114	0.024	0.541	**0.006**	0.049	0.003	0.454	**0.0033**
	All versus Sham	24 versus 12	**0.0002**	0.152	0.049	0.464	**0.001**	0.059	0.005	0.393	**0.0011**
											
Non AK only	Sham	11	Ref.	Ref.				Ref.			
	Ad35/Ad26	12	**0.0112**	0.161	0.033	0.784	**0.024**	0.0867	0.0058	0.7444	**0.0202**
	Ad35/Ad26/Env	12	**0.0002**					0.0326	0	0.2711	**0.0007**
	All versus Sham	24 versus 11	**0.0001**	0.076	0.016	0.378	**0.001**	0.0400	0.003	0.3137	**0.0005**
											
AK only	Sham	11	Ref.	Ref.				Ref.			
	Ad35/Ad26	12	0.446	0.368	0.032	4.252	0.424	0.425	0.006	9.423	0.932
	Ad35/Ad26/Env	12	0.697	0.682	0.091	5.133	0.710	0.904	0.055	14.949	1.000
	All versus Sham	24 versus 11	0.494	0.531	0.084	3.342	0.500	0.651	0.063	9.053	1.000

The bold entries indicate significant *P* values.

**Table 3:** Effects of Ad35/Ad26 and Ad35/Ad26/Env vaccination on the number of T/F variants divided into A/K or non-A/K genotypes in the SHD challenge study.

**Group**	**IRR for non-AK variants**				**IRR for AK variants**				***P*** value for equivalence test*
	**IRR**	**Lower 95%CI**	**Upper 95%CI**	***P*** **value**	**IRR**	**Lower 95%CI**	**Upper 95%CI**	***P*** **value**	
Sham	Ref.				Ref.				
Ad35/Ad26	0.171	0.080	0.365	**<0.001**	0.950	0.499	1.810	0.876	**<0.001**
Ad35/Ad26/Env	0.086	0.031	0.239	**<0.001**	0.450	0.202	1.002	**0.050**	**0.004**

IRR, incidence rate ratio.

^*^P value for testing equivalence of IRRs for AK and non-AK variants.
