# Supplementary material for: Adenovirus prime, Env protein boost vaccine protects against neutralization-resistant SIVsmE660 variants in rhesus monkeys
Source: Nat Commun. 2017 Jun 5;8:15740. doi: 10.1038/ncomms15740 (PMC5465370; doi:10.1038/ncomms15740)
Supplement: Supplementary Information — Supplementary Figures and Supplementary Tables [file ncomms15740-s1.pdf]

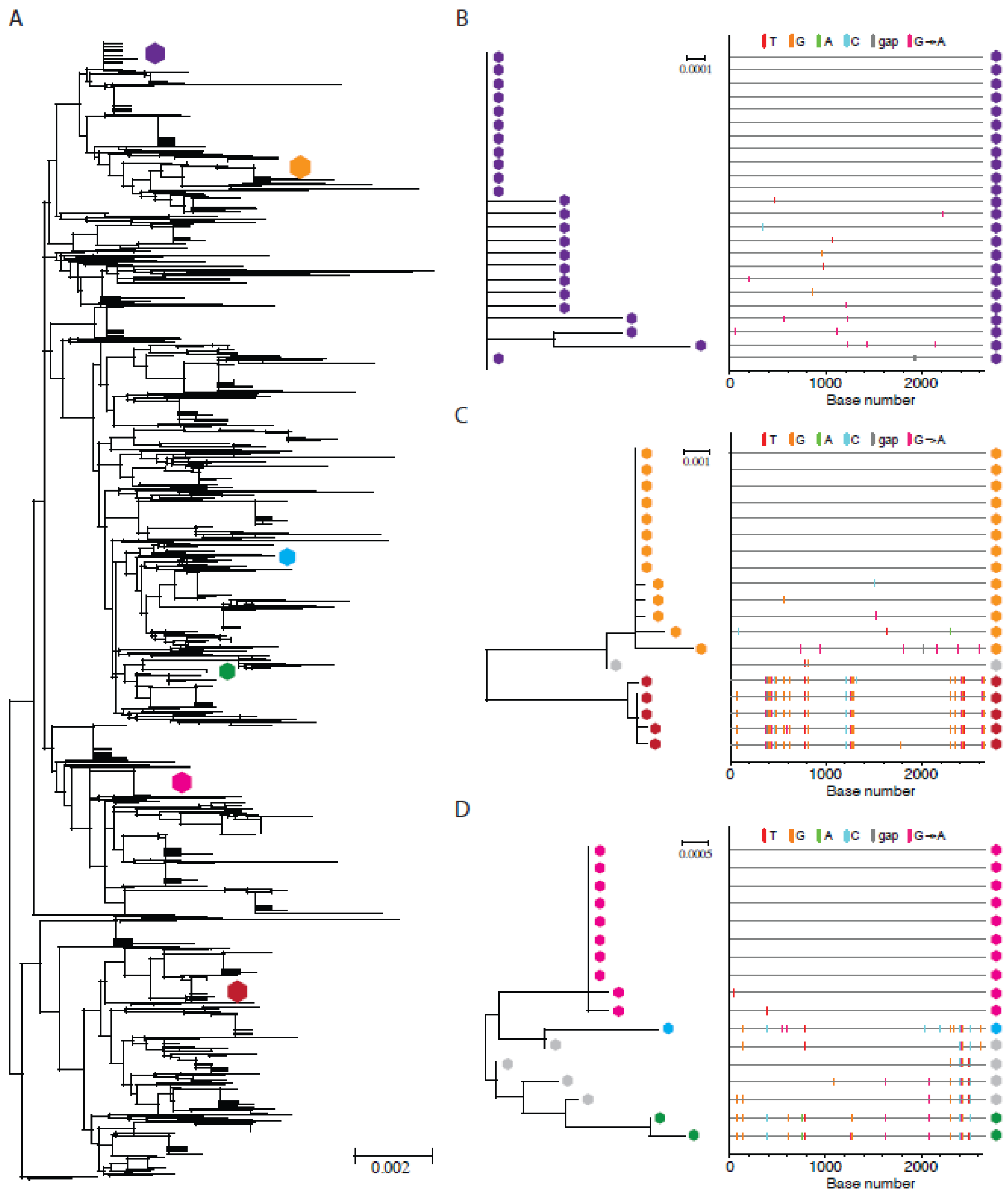

Supplementary Figure 1. Variant enumeration. Composite tree (A) and representative tree and highlighter alignments (B-D) demonstrate the ability to discriminate animals infected with a single T/F genomes (B), two TF variants (C) or three T/F variants (D). Each T/F lineage is color coded and mapped onto the composite tree. Recombinant genomes are identified phylogenetically and are shown in grey. Individual polymorphisms are shown as individual tick-marks in alignment.

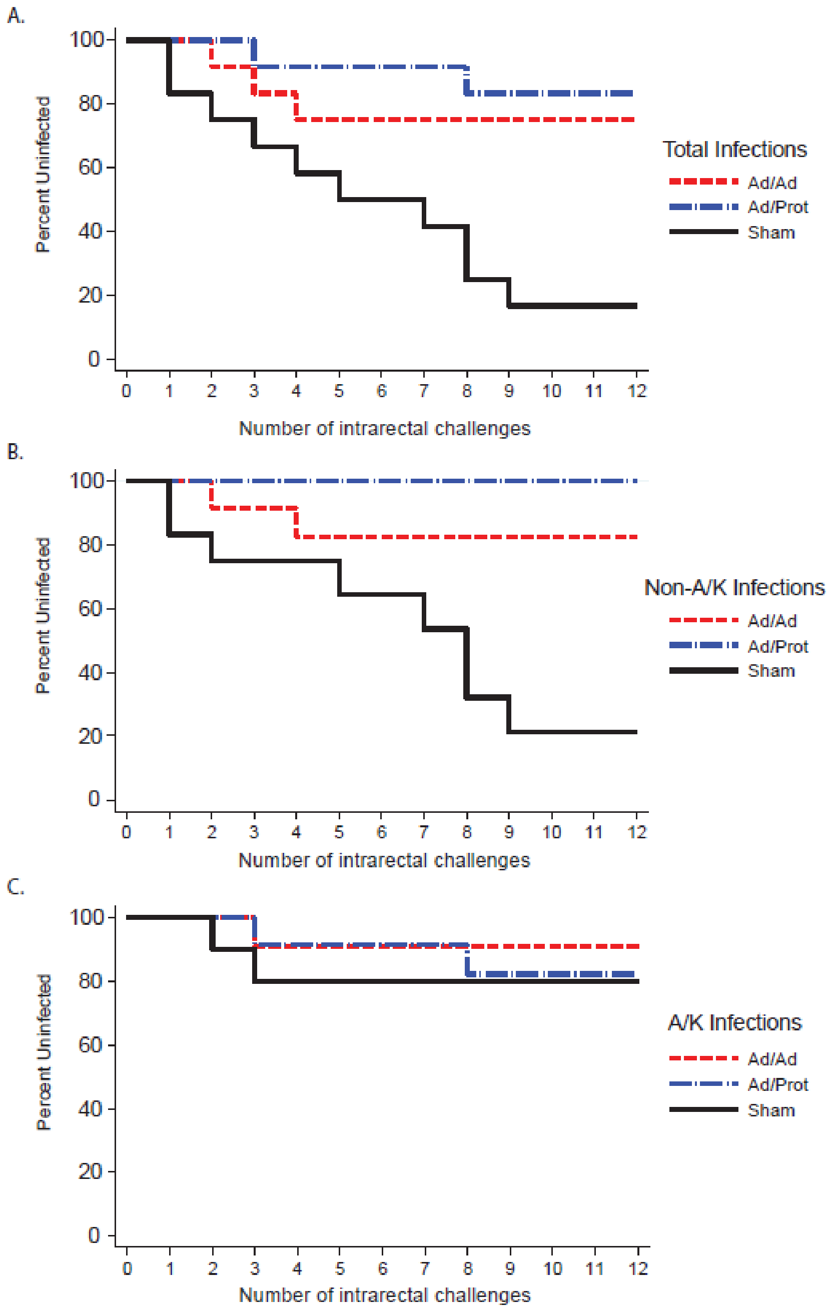

Supplementary Figure 2. Protective efficacy stratified by neutralization resistance in the RLD challenge study. The number of challenges required for detection of productive infection for each vaccine group separated into all T/F genomes (A), non-A/K (B), and A/K (C). Kaplan-Meier plots denote the percent of uninfected animals after each challenge. Ad/Env (blue) indicates animals primed with Ad35/Ad26 and boosted with adjuvanted Env gp140 (Ad35/Ad26/Env). Ad/Ad (red) indicates animals vaccinated with Ad35/Ad26 only.

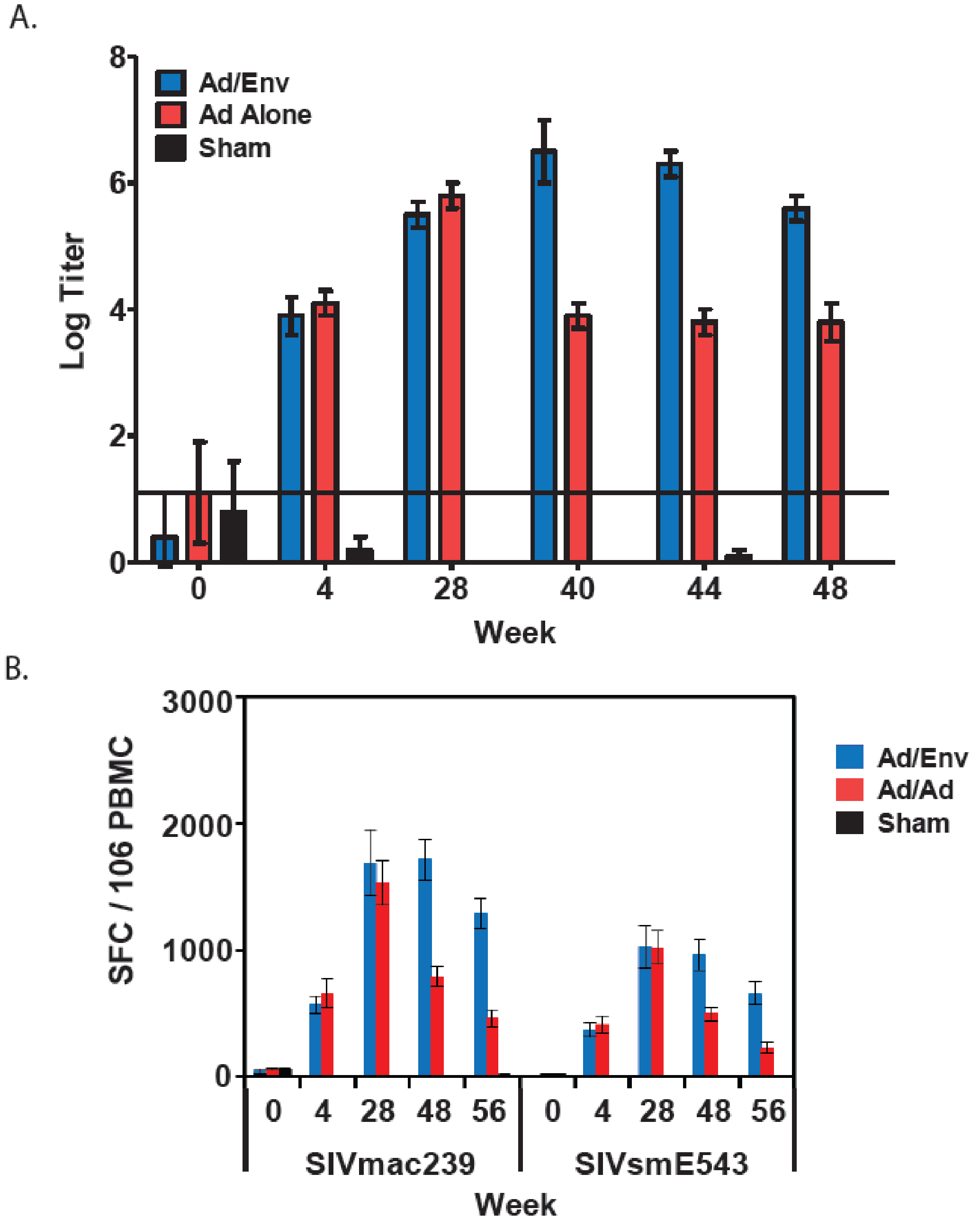

Supplementary Figure 3. Humoral and cellular immune responses in the SHD challenge study. Vaccine-induced binding antibodies titers (N=10/group) were determined by SIVmac251 Env ELISA at week 0, 4, 28, 40, 44, and 48 following vaccination (A). Cellular immune responses to SIVmac239 and SIVsmE543 Gag, Pol, and Env were determined by IFN-γ ELISPOT assay at weeks 0, 4, 28, 48, and 56 and reported as spot-forming cells (SFC) per million peripheral blood mononuclear cells (PBMC). Ad/Env (blue) indicates animals primed with Ad35/Ad26 and boosted with adjuvanted Env gp140 (Ad35/Ad26/Env). Ad/Ad (red) indicates animals vaccinated with Ad35/Ad26 only. Sham controls (black) were vaccinated with Ad35/Ad26 expressing no relevant antigens. Error bars represent standard error of mean (SEM).

Supplementary Table 1. Immunologic data

Log ELISA Titer (RLD Challenge Study)

Ad/Env	Ad	Ad/Env	Ad	Ad/Env	Ad	Ad/Env	Ad	Ad/Env	Ad	Ad/Env	Ad
Wk 0	Wk 0	Wk 4	Wk 4	Wk 28	Wk 28	Wk 40	Wk 40	Wk 44	Wk 44	Wk 48	Wk 48
0.0	0.0	4.3	4.8	5.8	5.3	6.3	4.8	6.3	5.3	5.8	4.8
0.0	0.0	4.3	4.3	5.8	5.3	6.3	5.3	5.8	5.3	6.3	5.3
0.0	0.0	4.3	4.8	5.8	5.3	6.7	4.8	5.8	5.3	6.3	4.8
0.0	0.0	4.3	4.3	5.8	5.8	6.7	4.8	5.8	5.3	6.7	5.3
0.0	0.0	3.8	3.8	5.8	5.3	6.7	4.8	5.8	4.8	6.3	4.8
0.0	0.0	4.3	3.8	5.8	5.3	6.3	4.8	5.8	5.3	5.8	4.8
0.0	0.0	3.8	4.3	5.8	5.8	6.3	5.3	6.3	5.3	6.3	5.3
0.0	0.0	4.3	4.3	5.8	5.3	6.3	4.8	5.8	4.8	6.7	4.8
0.0	0.0	4.3	4.3	5.3	5.8	5.8	4.8	5.8	5.3	5.3	5.3
0.0	0.0	4.3	4.3	6.3	5.8	6.3	5.3	5.8	5.8	5.3	5.3
0.0	0.0	3.8	3.8	5.3	5.3	5.8	5.3	5.8	5.3	5.8	5.3
0.0	0.0	4.3	4.3	6.7	5.3	6.3	5.3	5.8	4.8	6.3	5.3

Log ELISA Titer (SHD Challenge Study)

Ad/Env	Ad	Ad/Env	Ad	Ad/Env	Ad	Ad/Env	Ad	Ad/Env	Ad	Ad/Env	Ad
Wk 0	Wk 0	Wk 4	Wk 4	Wk 28	Wk 28	Wk 40	Wk 40	Wk 44	Wk 44	Wk 48	Wk 48
0.0	1.4	3.8	3.8	5.3	5.8	6.7	3.8	6.7	3.8	5.8	3.8
1.4	1.4	3.8	3.8	5.8	6.3	6.7	3.8	6.3	3.8	5.8	3.8
0.0	1.4	3.8	4.8	5.8	5.3	6.3	3.8	6.3	3.8	5.3	3.8
0.0	0.0	4.3	4.3	5.3	5.3	6.7	3.8	6.3	3.8	6.3	3.8
1.4	1.4	4.3	3.8	5.8	5.8	5.8	3.8	5.8	4.3	5.3	3.8
0.0	1.4	3.8	4.3	5.8	5.8	6.3	4.8	6.3	4.3	5.8	4.8
0.0	1.4	3.8	4.3	5.3	5.3	6.3	3.8	6.3	3.8	5.3	3.8
0.0	0.0	3.8	3.8	5.8	5.8	5.8	3.8	5.8	3.8	5.3	3.8
0.0	1.4	3.8	3.8	5.3	5.3	6.7	4.3	6.3	3.8	5.8	4.3
1.4	1.4	3.8	4.3	5.3	6.3	6.7	3.8	6.7	3.8	5.8	3.8

Raw ELISPOT Data (Spot-Forming Cells / 200,000 PBMC)
Week 0

Ad/Ad

Median												
R10	SIVmac239 Gag	SIVmac239 Env1	SIVmac239 Env2	SIVmac239 Pol1	SIVmac239 Pol2	SIVsm543 Gag	SIVsm543 Env1	SIVsm543 Env2	SIVsm543 Pol1	SIVsm543 Pol2	PHAM	
2	0	0	2	2	2	1	2	1	1	1	903	218-10
3	5	4	6	13	12	4	3	2	1	5	476	220-10
2	2	2	1	5	6	2	0	2	2	2	495	222-10
1	1	1	3	4	3	2	1	2	2	2	468	223-10
4	4	5	4	5	5	2	2	2	2	5	471	226-10
3	1	2	1	6	7	2	2	2	0	1	389	227-10
2	1	2	3	11	16	2	3	3	1	6	499	229-10
1	2	3	1	9	3	1	2	2	3	2	392	239-10
3	1	4	3	11	3	2	1	7	0	1	616	244-10
4	2	2	4	4	1	5	4	4	3	4	540	245-10

	Data											
	R10	SIVmac239 Gag	SIVmac239 Env1	SIVmac239 Env2	SIVmac239 Pol1	SIVmac239 Pol2	SIVsm543 Gag	SIVmac239 Env1	SIVsm543 Env2	SIVsm543 Pol1	SIVsm543 Pol2	PHAM
218-10	4	0	0	1	2	2	1	1	0	1	1	889
	0	0	0	3	2	1	0	2	2	1	0	917
220-10	3	2	3	5	17	9	3	5	1	1	4	477
	2	8	4	6	9	14	5	0	2	1	6	474
222-10	2	1	0	2	6	1	3	0	2	2	1	513
	1	3	3	0	4	10	1	0	1	1	3	476
223-10	2	0	2	0	2	4	4	2	1	2	3	457
	0	2	0	5	5	1	0	0	3	2	1	479
226-10	4	2	6	3	4	4	1	3	0	1	2	477
	3	5	4	5	6	5	2	1	3	2	8	464
227-10	4	1	1	1	7	6	1	2	2	0	0	384
	1	1	2	1	5	8	2	2	1	0	1	394
229-10	3	1	3	5	10	17	2	2	1	0	6	520
	0	0	1	1	12	14	1	4	4	2	5	478
239-10	2	3	2	1	9	2	0	1	3	2	1	388
	0	0	3	0	8	3	1	2	1	3	2	396
244-10	2	0	5	1	4	2	2	1	7	0	0	636
	3	2	2	5	17	3	2	0	6	0	1	596
245-10	3	2	3	6	1	2	3	5	4	1	4	541
	4	2	1	2	6	0	7	3	4	5	4	538

Ad/Env

Median												
R10	SIVmac239 Gag	SIVmac239 Env1	SIVmac239 Env2	SIVmac239 Pol1	SIVmac239 Pol2	SIVsm543 Gag	SIVsm543 Env1	SIVsm543 Env2	SIVsm543 Pol1	SIVsm543 Pol2	PHAM	
2	4	5	5	11	8	2	1	1	2	1	551	219-10
4	5	3	2	18	8	2	2	2	2	3	535	221-10
1	4	25	3	53	16	2	2	5	2	1	777	225-10
4	1	1	1	13	12	1	1	2	3	2	417	232-10
1	4	4	2	4	3	2	1	3	2	1	337	233-10
3	4	2	3	2	4	3	5	3	1	4	541	234-10
1	1	2	2	17	1	2	14	1	0	2	578	235-10
2	2	4	5	55	17	2	4	2	2	3	372	231-10
6	5	4	5	9	5	2	6	8	3	3	574	240-10
4	2	2	2	2	1	2	4	3	3	1	556	246-10

	Data											
	R10	SIVmac239 Gag	SIVmac239 Env1	SIVmac239 Env2	SIVmac239 Pol1	SIVmac239 Pol2	SIVsm543 Gag	SIVmac239 Env1	SIVsm543 Env2	SIVsm543 Pol1	SIVsm543 Pol2	PHAM
219-10	2	4	2	5	10	8	2	1	1	2	2	565
	2	4	7	4	12	7	1	1	1	1	0	536
221-10	4	1	3	2	9	9	2	3	0	1	1	523
	3	9	2	1	27	7	1	0	3	2	4	546
225-10	1	4	0	4	70	13	1	1	2	2	1	829
	1	3	49	2	36	19	3	3	7	1	0	725
232-10	3	1	1	1	16	11	0	1	2	3	3	448
	5	1	1	1	9	12	1	0	1	3	1	386
233-10	1	1	4	0	4	3	4	1	3	2	1	340
	1	6	3	4	3	2	0	0	2	1	0	333
234-10	3	5	2	3	1	4	2	5	3	0	4	558
	2	2	1	2	3	3	3	5	2	2	3	524
235-10	1	2	2	3	24	2	1	26	2	0	2	584
	1	0	1	0	10	0	3	1	0	0	1	572
231-10	3	2	5	2	65	21	0	4	0	1	3	356
	0	2	2	7	45	13	3	3	3	2	3	388
240-10	11	7	7	2	13	7	1	8	9	3	2	582
	1	3	1	7	5	2	3	4	6	2	4	565
246-10	5	3	4	2	2	1	2	3	4	2	0	560
	3	1	0	2	1	1	1	4	2	3	1	552

Sham

Median												
R10	SIVmac239 Gag	SIVmac239 Env1	SIVmac239 Env2	SIVmac239 Pol1	SIVmac239 Pol2	SIVsm543 Gag	SIVsm543 Env1	SIVsm543 Env2	SIVsm543 Pol1	SIVsm543 Pol2	PHAM	
2	1	2	1	4	1	0	1	0	1	0	261	217-10
6	4	2	4	48	47	2	4	6	3	2	498	224-10
2	1	1	1	2	3	3	0	3	1	2	482	228-10
8	5	7	3	20	20	4	4	2	5	9	599	230-10
1	4	1	0	1	3	3	9	1	2	0	490	236-10
4	2	2	2	1	2	3	1	2	4	2	393	237-10
2	0	0	2	2	2	2	2	1	2	2	526	238-10
2	2	1	1	1	1	1	1	3	2	1	470	241-10
2	1	2	1	0	1	3	0	2	3	3	479	242-10
2	3	4	1	6	3	3	4	3	3	3	605	243-10

	Data											
	R10	SIVmac239 Gag	SIVmac239 Env1	SIVmac239 Env2	SIVmac239 Pol1	SIVmac239 Pol2	SIVsm543 Gag	SIVmac239 Env1	SIVsm543 Env2	SIVsm543 Pol1	SIVsm543 Pol2	PHAM
217-10	2	1	2	0	6	1	0	1	0	1	0	269
	1	1	1	1	2	1	0	0	0	0	0	252
224-10	3	5	1	6	54	47	3	3	11	3	4	526
	8	3	2	2	42	46	0	4	1	3	0	469
228-10	1	1	1	0	2	5	1	0	3	0	3	502
	2	0	1	1	1	1	4	0	2	1	0	462
230-10	9	7	8	1	29	19	4	3	3	5	16	597
	6	2	5	4	10	20	3	5	1	4	2	601
236-10	0	3	2	0	2	4	3	17	0	0	0	483
	1	5	0	0	0	1	2	1	1	4	0	497
237-10	2	1	2	1	0	2	4	1	3	1	3	389
	5	3	2	3	1	2	2	1	1	6	0	397
238-10	1	0	0	2	2	3	1	2	0	1	1	515
	3	0	0	1	1	0	2	2	2	2	3	537
241-10	2	0	0	1	1	0	2	0	2	2	0	464
	1	3	1	1	1	2	0	1	3	2	1	476
242-10	2	0	2	1	0	1	4	0	0	3	4	481
	1	2	2	1	0	1	2	0	3	2	1	477
243-10	0	5	6	1	6	3	3	6	2	3	2	628
	3	1	1	1	6	3	2	1	3	2	4	581

Raw ELISPOT Data (Spot-Forming Cells / 200,000 PBMC)
Week 4

Ad/Ad

Median

R10	SIVmac239 Gag	SIVmac239 Env1	SIVmac239 Env2	SIVmac239 Pol1	SIVmac239 Pol2	SIVsm543 Gag	SIVsm543 Env1	SIVsm543 Env2	SIVsm543 Pol1	SIVsm543 Pol2	PHAM	
0	146	17	14	12	7	131	6	4	20	5	713	218-10
1	15	65	3	71	5	14	26	12	43	0	488	220-10
1	30	165	10	46	93	27	14	11	44	21	456	222-10
1	129	13	17	5	3	89	4	9	2	1	448	223-10
0	13	9	7	1	1	17	5	2	3	1	203	226-10
1	71	22	11	5	7	69	12	13	1	4	389	227-10
1	10	13	15	8	14	9	8	18	4	4	548	229-10
1	23	13	6	11	5	10	7	8	7	4	383	239-10
0	13	8	8	7	1	8	2	3	8	2	382	244-10
14	76	46	32	28	34	63	34	28	15	36	448	245-10

Data

	R10	SIVmac239 Gag	SIVmac239 Env1	SIVmac239 Env2	SIVmac239 Pol1	SIVmac239 Pol2	SIVsm543 Gag	SIVmac239 Env1	SIVsm543 Env2	SIVsm543 Pol1	SIVsm543 Pol2	PHAM
218-10	0	135	16	12	14	6	128	8	3	15	3	693
	0	157	18	15	10	8	134	3	4	24	7	733
220-10	1	15	63	1	61	1	18	35	13	45	0	488
	0	15	67	5	81	9	9	17	11	41	0	487
222-10	1	23	154	10	42	98	27	13	9	49	14	469
	1	37	176	9	49	87	27	15	13	38	27	442
223-10	0	121	13	17	6	3	98	4	11	3	1	454
	1	137	12	16	3	3	79	4	7	1	1	442
226-10	0	11	10	5	1	1	20	6	2	2	1	147
	0	15	7	9	0	1	13	3	1	3	1	258
227-10	1	66	15	10	5	6	67	11	13	2	6	393
	1	76	29	11	4	8	71	13	12	0	2	385
229-10	1	8	15	17	10	14	8	7	17	4	2	531
	1	12	11	13	5	13	10	8	18	4	5	564
239-10	0	23	10	7	10	6	9	3	10	5	7	421
	1	22	15	5	11	4	11	10	6	9	1	345
244-10	0	9	8	10	6	1	7	1	2	6	1	347
	0	17	8	6	8	1	9	3	3	10	3	417
245-10	14	64	39	37	23	39	60	32	27	17	28	468
	14	88	52	26	33	29	66	35	28	13	43	428

Ad/Env

Median

R10	SIVmac239 Gag	SIVmac239 Env1	SIVmac239 Env2	SIVmac239 Pol1	SIVmac239 Pol2	SIVsm543 Gag	SIVsm543 Env1	SIVsm543 Env2	SIVsm543 Pol1	SIVsm543 Pol2	PHAM	
1	23	16	11	5	8	11	5	19	7	13	549	219-10
1	42	63	41	32	12	24	31	34	14	5	557	221-10
2	39	55	12	6	5	47	20	4	8	6	511	225-10
1	73	16	17	44	15	73	2	13	45	8	555	232-10
1	13	19	31	9	4	10	14	16	9	12	507	233-10
2	143	35	58	13	47	129	7	29	4	33	601	234-10
2	7	16	30	6	5	4	4	6	5	4	468	235-10
0	14	15	11	28	21	12	6	13	22	13	367	231-10
6	32	21	18	24	10	19	5	3	15	2	552	240-10
3	6	11	4	4	5	4	3	4	6	2	449	246-10

Data

	R10	SIVmac239 Gag	SIVmac239 Env1	SIVmac239 Env2	SIVmac239 Pol1	SIVmac239 Pol2	SIVsm543 Gag	SIVmac239 Env1	SIVsm543 Env2	SIVsm543 Pol1	SIVsm543 Pol2	PHAM
219-10	0	16	15	8	6	7	10	5	15	6	11	554
	1	29	16	13	4	9	11	5	22	7	15	543
221-10	0	41	63	37	37	17	27	34	41	10	3	576
	1	42	63	45	26	6	21	28	26	18	6	537
225-10	1	37	66	13	8	5	48	20	4	7	8	528
	2	40	43	11	3	4	46	20	4	8	4	494
232-10	1	84	20	18	41	13	82	2	12	50	7	578
	0	61	12	15	47	16	64	2	13	40	8	532
233-10	1	14	11	23	3	2	5	9	14	14	11	496
	0	12	26	38	14	6	15	19	17	4	13	517
234-10	3	156	39	53	16	37	118	6	29	2	32	601
	1	130	30	63	10	56	140	7	29	6	34	601
235-10	0	8	15	30	9	7	4	2	2	0	4	457
	3	6	17	29	2	3	4	6	9	9	3	479
231-10	0	10	4	13	15	17	10	5	16	20	9	342
	0	18	26	9	40	24	13	7	9	23	17	392
240-10	8	29	15	13	16	9	16	7	3	14	2	569
	3	35	27	22	32	10	21	2	3	15	2	534
246-10	5	7	15	3	8	2	3	4	4	2	3	457
	1	4	7	4	0	7	4	2	3	9	1	440

Sham

Median

R10	SIVmac239 Gag	SIVmac239 Env1	SIVmac239 Env2	SIVmac239 Pol1	SIVmac239 Pol2	SIVsm543 Gag	SIVsm543 Env1	SIVsm543 Env2	SIVsm543 Pol1	SIVsm543 Pol2	PHAM	
0	1	1	1	1	0	1	2	0	3	0	46	217-10
1	1	1	1	0	2	0	0	0	1	1	409	224-10
1	1	0	0	0	1	0	0	0	1	1	263	228-10
1	3	6	2	1	1	1	4	0	1	1	414	230-10
1	1	1	0	0	1	0	1	1	0	0	420	236-10
0	0	0	1	2	1	0	0	1	1	0	392	237-10
0	1	0	1	0	2	1	1	1	1	1	218	238-10
0	1	1	0	1	0	0	0	0	0	1	183	241-10
0	0	1	0	1	0	1	0	0	1	1	332	242-10
2	2	1	1	0	0	1	0	1	1	0	349	243-10

Data

	R10	SIVmac239 Gag	SIVmac239 Env1	SIVmac239 Env2	SIVmac239 Pol1	SIVmac239 Pol2	SIVsm543 Gag	SIVmac239 Env1	SIVsm543 Env2	SIVsm543 Pol1	SIVsm543 Pol2	PHAM
217-10	0	1	1	0	1	0	1	2	0	2	0	38
	0	0	1	1	0	0	0	1	0	4	0	54
224-10	1	0	0	0	0	2	0	0	0	1	1	408
	1	1	2	1	0	1	0	0	0	1	0	410
228-10	2	0	0	0	0	2	0	0	0	2	0	235
	0	1	0	0	0	0	0	0	0	0	1	291
230-10	1	4	5	1	1	0	0	4	0	1	1	412
	0	1	6	3	0	2	1	3	0	0	1	416
236-10	1	1	1	0	0	1	0	1	0	0	0	400
	1	0	0	0	0	1	0	1	1	0	0	439
237-10	0	0	0	0	0	1	0	0	1	0	0	355
	0	0	0	1	4	0	0	0	1	1	0	428
238-10	0	1	0	0	0	3	0	1	0	1	0	193
	0	0	0	1	0	0	1	0	1	0	1	242
241-10	0	1	0	0	0	0	0	0	0	0	1	179
	0	0	1	0	1	0	0	0	0	0	0	187
242-10	0	0	0	0	1	0	0	0	0	0	1	389
	0	0	1	0	0	0	1	0	0	1	0	275
243-10	1	3	0	1	0	0	2	0	1	0	0	345
	3	0	1	1	0	0	0	0	0	1	0	353

Raw ELISPOT Data (Spot-Forming Cells / 200,000 PBMC)
Week 28

Ad/Ad

Median													Data											
R10	SIVmac239 Gag	SIVmac239 Env1	SIVmac239 Env2	SIVmac239 Pol1	SIVmac239 Pol2	SIVsm543 Gag	SIVsm543 Env1	SIVsm543 Env2	SIVsm543 Pol1	SIVsm543 Pol2	PHAM		R10	SIVmac239 Gag	SIVmac239 Env1	SIVmac239 Env2	SIVmac239 Pol1	SIVmac239 Pol2	SIVsm543 Gag	SIVmac239 Env1	SIVsm543 Env2	SIVsm543 Pol1	SIVsm543 Pol2	PHAM
1	198	38	26	48	17	178	4	22	32	11	*	218-10	1	192	37	28	52	11	167	3	21	30	5	*
													1	203	39	23	44	22	189	4	22	34	17	*
0	66	278	2	230	9	17	148	13	201	1	*	220-10	0	69	283	2	224	7	15	156	12	192	0	*
													0	63	273	2	236	10	18	139	13	209	1	*
1	22	185	6	46	116	26	15	1	30	8	*	222-10	1	23	190	7	41	118	33	22	0	30	8	*
													0	20	180	5	51	113	18	7	1	29	8	*
1	156	30	32	43	16	137	12	23	41	10	*	223-10	0	156	32	31	38	18	153	12	20	45	5	*
													1	155	28	33	48	14	121	11	26	37	14	*
1	76	45	13	12	7	62	19	15	5	11	*	226-10	0	75	42	10	16	3	55	17	12	7	8	*
													1	77	48	16	7	10	68	20	17	3	13	*
17	121	77	57	38	22	91	42	32	17	20	*	227-10	8	120	85	50	39	16	92	42	38	17	14	*
													26	121	69	64	36	28	89	42	26	16	26	*
1	18	26	47	24	41	13	8	44	17	9	*	229-10	0	16	25	42	20	51	8	7	36	16	8	*
													1	20	26	51	27	30	18	8	52	18	9	*
1	15	21	43	19	8	9	7	14	18	1	*	239-10	0	14	19	42	16	11	9	6	13	23	1	*
													1	16	23	44	21	5	8	7	14	12	1	*
1	45	47	48	34	5	31	34	76	17	2	*	244-10	1	49	47	40	30	3	27	33	82	18	1	*
													0	40	47	56	37	7	34	35	70	16	2	*
0	72	45	57	17	68	73	27	9	3	59	*	245-10	0	79	44	66	20	71	83	31	10	4	52	*
													0	64	45	47	13	65	62	23	7	1	66	*

Ad/Env

Median													Data											
R10	SIVmac239 Gag	SIVmac239 Env1	SIVmac239 Env2	SIVmac239 Pol1	SIVmac239 Pol2	SIVsm543 Gag	SIVsm543 Env1	SIVsm543 Env2	SIVsm543 Pol1	SIVsm543 Pol2	PHAM		R10	SIVmac239 Gag	SIVmac239 Env1	SIVmac239 Env2	SIVmac239 Pol1	SIVmac239 Pol2	SIVsm543 Gag	SIVmac239 Env1	SIVsm543 Env2	SIVsm543 Pol1	SIVsm543 Pol2	PHAM
1	15	25	21	8	15	11	6	23	11	9	*	219-10	0	14	27	23	7	16	12	4	20	10	13	*
													1	15	23	18	9	13	9	7	26	12	4	*
2	185	163	364	57	48	138	99	43	26	46	*	221-10	2	190	182	352	60	56	146	117	44	30	51	*
													2	179	143	375	54	39	129	80	42	22	41	*
0	70	84	16	33	51	84	50	17	23	40	*	225-10	0	68	91	20	29	45	80	46	24	21	30	*
													0	72	77	12	37	57	87	53	9	25	49	*
0	173	19	41	72	8	198	4	12	50	17	*	232-10	0	192	16	48	81	8	192	4	12	53	19	*
													0	154	21	34	63	8	203	4	12	46	14	*
1	16	7	23	18	12	5	5	2	8	4	*	233-10	2	12	6	24	17	8	3	5	0	5	4	*
													0	19	7	22	18	15	6	5	3	10	4	*
0	336	59	145	77	308	320	22	43	42	201	*	234-10	0	331	57	163	79	310	335	22	44	37	195	*
													0	340	60	127	75	306	304	22	41	46	207	*
1	32	73	62	47	18	22	35	48	20	12	*	235-10	1	27	80	67	53	9	14	24	48	21	12	*
													0	37	65	56	40	26	29	45	48	18	12	*
1	24	28	35	88	70	30	19	15	14	52	*	231-10	0	26	31	37	100	68	30	20	13	15	61	*
													1	22	24	32	76	72	30	17	16	13	42	*
2	118	67	31	60	13	96	18	19	34	2	*	240-10	2	124	64	34	55	14	103	18	23	37	0	*
													2	111	70	27	65	11	88	18	14	30	4	*
0	51	222	26	35	41	27	17	6	30	8	*	246-10	0	54	213	25	40	42	32	18	6	35	6	*
													0	47	230	27	29	39	21	15	5	25	10	*

Sham

Median													Data											
R10	SIVmac239 Gag	SIVmac239 Env1	SIVmac239 Env2	SIVmac239 Pol1	SIVmac239 Pol2	SIVsm543 Gag	SIVsm543 Env1	SIVsm543 Env2	SIVsm543 Pol1	SIVsm543 Pol2	PHAM		R10	SIVmac239 Gag	SIVmac239 Env1	SIVmac239 Env2	SIVmac239 Pol1	SIVmac239 Pol2	SIVsm543 Gag	SIVmac239 Env1	SIVsm543 Env2	SIVsm543 Pol1	SIVsm543 Pol2	PHAM
1	1	1	1	1	0	1	0	0	1	1	*	217-10	1	0	1	1	1	0	1	0	0	1	1	*
													1	1	1	0	0	0	0	0	0	0	0	*
1	1	0	1	2	1	1	1	0	1	0	*	224-10	1	0	0	2	2	1	1	1	0	0	0	*
													0	1	0	0	2	0	0	0	0	2	0	*
1	0	1	1	1	1	1	0	0	0	0	*	228-10	0	0	0	1	0	0	1	0	0	0	0	*
													2	0	1	0	1	1	0	0	0	0	0	*
1	1	3	1	5	2	2	3	0	0	2	*	230-10	1	1	3	0	3	1	1	1	0	0	1	*
													1	1	2	1	6	2	2	4	0	0	3	*
1	4	2	2	2	2	4	3	1	3	0	*	236-10	1	5	1	3	0	2	2	2	0	4	0	*
													0	2	3	0	3	2	5	4	1	1	0	*
1	2	1	1	4	1	1	0	0	1	1	*	237-10	1	2	1	0	3	1	0	0	0	1	1	*
													0	1	0	1	5	0	1	0	0	0	1	*
0	1	1	1	3	1	1	0	1	0	0	*	238-10	0	0	0	0	0	0	1	0	0	0	0	*
													0	2	1	1	5	1	0	0	1	0	0	*
1	1	1	0	1	1	2	1	2	0	1	*	241-10	1	1	0	0	1	0	2	1	1	0	0	*
													1	0	1	0	1	2	2	1	2	0	1	*
0	1	1	0	1	0	0	1	1	1	1	*	242-10	0	0	0	0	1	0	0	0	0	1	0	*
													0	1	1	0	1	0	0	1	1	1	1	*
1	2	0	0	1	1	0	0	0	0	0	*	243-10	2	2	0	0	0	0	0	0	0	0	0	*
													0	1	0	0	1	1	0	0	0	0	0	*

Raw ELISPOT Data (Spot-Forming Cells / 200,000 PBMC)
Week 48

Ad/Ad

Median											
R10	SIVmac239 Gag	SIVmac239 Env1	SIVmac239 Env2	SIVmac239 Pol1	SIVmac239 Pol2	SIVsm543 Gag	SIVsm543 Env1	SIVsm543 Env2	SIVsm543 Pol1	SIVsm543 Pol2	PHAM
1	71	13	14	17	5	63	5	8	12	6	272
0	24	114	5	100	4	9	64	4	65	2	191
1	11	87	8	25	80	5	8	1	24	10	185
4	40	8	8	12	4	31	7	7	8	3	264
3	44	27	8	10	5	30	12	16	4	7	250
2	68	57	37	13	18	75	15	27	9	7	183
2	5	16	20	9	15	6	3	16	4	6	275
0	12	11	7	16	4	6	2	4	6	1	195
1	12	9	20	12	7	4	6	14	2	0	265
2	61	22	38	24	58	42	18	3	6	33	291

	Data											
	R10	SIVmac239 Gag	SIVmac239 Env1	SIVmac239 Env2	SIVmac239 Pol1	SIVmac239 Pol2	SIVsm543 Gag	SIVmac239 Env1	SIVsm543 Env2	SIVsm543 Pol1	SIVsm543 Pol2	PHAM
218-10	1	80	13	11	19	4	62	3	11	13	6	265
	0	62	12	17	14	6	64	6	5	10	5	279
220-10	0	31	117	2	103	4	8	63	5	59	1	207
	0	16	111	8	96	3	9	65	2	70	2	174
222-10	2	12	92	11	30	96	4	7	0	31	9	176
	0	10	82	5	19	63	6	8	2	16	11	194
223-10	1	43	7	9	10	4	32	4	5	2	4	275
	7	37	8	6	13	4	30	9	9	14	1	252
226-10	2	48	31	9	13	7	29	13	19	5	7	255
	3	39	22	7	6	2	31	11	13	3	6	245
227-10	1	69	66	39	14	20	85	14	29	6	5	185
	2	67	48	35	11	16	64	15	24	11	9	180
229-10	1	6	15	18	7	16	5	1	10	3	4	279
	2	3	16	22	11	13	7	4	22	4	7	271
239-10	0	7	9	6	11	1	6	1	4	5	0	214
	0	16	12	7	21	6	6	3	3	6	1	175
244-10	0	10	8	25	9	7	4	6	14	3	0	294
	1	14	10	15	14	6	4	6	13	0	0	235
245-10	2	62	19	31	26	64	36	22	4	7	33	307
	1	60	25	45	22	52	48	13	1	4	33	274

Ad/Env

Median											
R10	SIVmac239 Gag	SIVmac239 Env1	SIVmac239 Env2	SIVmac239 Pol1	SIVmac239 Pol2	SIVsm543 Gag	SIVsm543 Env1	SIVsm543 Env2	SIVsm543 Pol1	SIVsm543 Pol2	PHAM
1	14	94	58	10	11	5	14	83	6	6	333
1	90	75	103	38	23	47	25	54	23	26	312
0	10	56	32	4	7	13	36	25	6	3	262
2	89	60	51	34	12	86	2	31	21	6	247
0	6	15	113	11	5	5	4	17	3	2	122
4	151	189	260	45	120	155	17	178	19	73	268
1	10	59	65	16	13	10	10	46	7	7	295
1	15	84	75	75	44	18	18	50	7	36	227
5	55	125	156	43	6	51	13	147	18	3	293
2	27	163	53	17	21	10	46	42	13	7	313

	Data											
	R10	SIVmac239 Gag	SIVmac239 Env1	SIVmac239 Env2	SIVmac239 Pol1	SIVmac239 Pol2	SIVsm543 Gag	SIVmac239 Env1	SIVsm543 Env2	SIVsm543 Pol1	SIVsm543 Pol2	PHAM
219-10	2	11	87	48	11	9	4	11	87	9	5	334
	0	17	101	67	8	12	6	16	79	2	6	331
221-10	0	80	78	83	28	23	58	31	62	23	26	316
	1	99	72	122	47	22	35	19	46	23	26	307
225-10	0	13	60	33	2	5	16	46	18	5	2	270
	0	7	52	31	6	9	9	25	32	7	4	254
232-10	4	97	54	53	26	13	81	1	29	25	10	238
	0	81	65	49	41	11	91	3	32	17	2	255
233-10	0	5	15	124	14	4	6	3	20	6	2	116
	0	6	14	101	8	5	3	5	14	0	1	127
234-10	1	160	176	239	44	108	149	14	171	19	74	295
	7	141	202	280	46	132	161	19	184	18	72	241
235-10	0	10	55	74	19	10	9	10	40	9	3	302
	1	9	63	56	12	16	10	10	52	5	10	288
231-10	0	18	90	84	79	49	16	22	64	7	35	208
	2	11	77	66	71	39	20	14	35	7	37	246
240-10	4	65	141	164	49	7	50	12	146	12	1	289
	5	45	109	147	37	5	52	13	148	23	4	297
246-10	2	30	164	55	16	22	11	49	47	14	3	335
	1	23	162	50	18	19	8	43	36	12	11	291

Sham

Median											
R10	SIVmac239 Gag	SIVmac239 Env1	SIVmac239 Env2	SIVmac239 Pol1	SIVmac239 Pol2	SIVsm543 Gag	SIVsm543 Env1	SIVsm543 Env2	SIVsm543 Pol1	SIVsm543 Pol2	PHAM
2	2	2	3	22	4	2	1	2	1	1	298
5	1	2	1	21	6	1	1	1	1	1	417
0	0	2	6	0	1	2	0	1	2	1	301
1	1	4	4	3	1	1	6	0	2	1	323
1	1	0	4	1	3	6	1	0	2	0	247
1	1	1	1	4	1	0	2	0	0	0	289
1	1	2	1	3	2	1	0	1	0	0	285
1	2	2	3	2	2	0	3	1	1	1	247
1	2	1	2	9	34	1	1	1	1	0	338
1	1	0	2	8	5	1	1	1	1	0	320

	Data											
	R10	SIVmac239 Gag	SIVmac239 Env1	SIVmac239 Env2	SIVmac239 Pol1	SIVmac239 Pol2	SIVsm543 Gag	SIVmac239 Env1	SIVsm543 Env2	SIVsm543 Pol1	SIVsm543 Pol2	PHAM
217-10	0	0	1	2	19	4	1	1	1	0	0	304
	3	3	3	3	25	3	2	0	3	2	1	292
224-10	5	0	3	1	18	2	1	1	0	2	1	424
	4	1	0	0	24	9	0	1	1	0	1	410
228-10	0	0	0	5	0	0	2	0	0	1	0	304
	0	0	3	6	0	2	1	0	1	3	1	297
230-10	1	0	4	6	2	0	0	4	0	3	1	332
	0	1	3	2	3	1	2	7	0	0	0	313
236-10	0	0	0	3	0	0	5	1	0	3	0	298
	1	1	0	4	1	5	7	1	0	0	0	196
237-10	1	1	1	1	2	0	0	2	0	0	0	289
	1	1	1	0	5	1	0	2	0	0	0	289
238-10	1	0	0	1	4	2	1	0	0	0	0	297
	0	1	3	1	2	1	1	0	1	0	0	272
241-10	1	3	1	3	0	1	0	3	0	1	0	249
	0	0	3	2	4	3	0	2	2	1	1	245
242-10	0	2	1	1	10	0	1	1	0	0	0	352
	1	1	0	2	8	67	0	1	1	1	0	324
243-10	0	0	0	0	5	4	0	1	1	1	0	314
	1	1	0	3	11	5	1	0	0	0	0	325

Raw ELISPOT Data (Spot-Forming Cells / 200,000 PBMC)
Week 56

Ad/Ad

Median

Animal	R10	SIVmac239 Gag	SIVmac239 Env1	SIVmac239 Env2	SIVmac239 Pol1	SIVmac239 Pol2	SIVsm543 Gag	SIVsm543 Env1	SIVsm543 Env2	SIVsm543 Pol1	SIVsm543 Pol2	PHAM
218-10	2	49	11	6	19	5	44	1	5	6	3	685
220-10	1	32	113	5	120	8	5	68	2	67	0	450
222-10	0	9	55	3	23	55	7	0	1	9	2	449
223-10	2	39	7	4	9	6	32	4	9	9	2	526
226-10	0	29	9	8	3	2	23	9	8	2	3	599
227-10	0	38	12	16	6	4	24	7	5	0	3	393
229-10	1	2	7	10	4	9	2	1	11	3	4	533
239-10	0	5	9	6	6	0	4	2	3	5	1	274
244-10	2	25	15	19	21	4	5	6	18	2	2	483
245-10	1	36	13	19	9	28	26	7	8	2	27	515

Data

Animal	R10	SIVmac239 Gag	SIVmac239 Env1	SIVmac239 Env2	SIVmac239 Pol1	SIVmac239 Pol2	SIVsm543 Gag	SIVmac239 Env1	SIVsm543 Env2	SIVsm543 Pol1	SIVsm543 Pol2	PHAM
218-10	1	48	10	4	21	6	34	1	5	7	3	712
	2	50	12	8	16	4	54	1	5	5	3	658
220-10	1	27	108	4	115	5	3	63	2	59	0	434
	1	36	117	5	125	11	6	73	1	74	0	466
222-10	0	9	57	1	17	60	5	0	0	7	1	433
	0	9	53	4	29	50	9	0	1	11	2	465
223-10	3	34	9	3	8	8	35	5	12	8	1	538
	1	43	5	4	10	4	29	3	5	9	3	514
226-10	0	25	9	7	4	2	25	9	10	0	4	605
	0	32	8	8	1	2	20	8	5	3	2	593
227-10	0	41	14	16	5	3	22	7	6	0	3	392
	0	35	10	15	7	5	25	7	4	0	2	393
229-10	0	1	8	13	3	10	1	0	9	4	3	499
	1	2	6	6	4	8	3	2	12	2	4	567
239-10	0	2	4	7	10	0	4	3	3	6	1	317
	0	8	14	5	2	0	3	1	2	3	0	230
244-10	0	25	18	16	21	2	4	7	16	0	3	489
	4	24	11	22	20	6	5	5	20	3	1	476
245-10	0	37	11	21	12	27	31	4	7	3	24	527
	1	34	14	17	5	29	21	9	9	0	30	503

Ad/Env

Median

Animal	R10	SIVmac239 Gag	SIVmac239 Env1	SIVmac239 Env2	SIVmac239 Pol1	SIVmac239 Pol2	SIVsm543 Gag	SIVsm543 Env1	SIVsm543 Env2	SIVsm543 Pol1	SIVsm543 Pol2	PHAM
219-10	0	9	82	36	5	7	7	10	78	4	2	584
221-10	0	126	68	107	42	22	51	31	47	16	20	472
225-10	2	20	145	110	7	15	20	53	59	3	4	510
232-10	1	66	46	37	41	8	63	4	18	23	4	374
233-10	1	5	30	93	11	5	4	12	20	7	3	295
234-10	2	129	192	194	22	85	119	17	194	22	40	559
235-10	2	5	35	52	14	8	3	7	36	3	3	514
231-10	0	13	59	44	39	37	10	12	22	11	28	484
240-10	3	26	71	162	14	4	33	8	95	13	2	534
246-10	2	29	144	77	13	13	18	28	51	17	6	587

Data

Animal	R10	SIVmac239 Gag	SIVmac239 Env1	SIVmac239 Env2	SIVmac239 Pol1	SIVmac239 Pol2	SIVsm543 Gag	SIVmac239 Env1	SIVsm543 Env2	SIVsm543 Pol1	SIVsm543 Pol2	PHAM
219-10	0	12	92	29	7	7	7	9	85	4	0	563
	0	6	71	42	2	6	6	11	71	3	3	605
221-10	0	120	84	108	48	23	54	43	44	12	25	465
	0	131	51	105	36	21	47	19	49	19	14	479
225-10	2	15	132	102	4	19	14	46	64	1	3	530
	1	25	157	118	10	10	26	59	54	4	4	490
232-10	1	66	54	40	47	9	71	4	27	22	5	387
	0	65	37	33	34	6	55	4	9	24	3	361
233-10	0	6	26	92	8	1	4	12	15	10	2	318
	1	4	34	94	14	9	4	11	24	4	3	272
234-10	2	136	206	178	18	80	126	15	195	29	47	576
	2	121	178	209	25	89	111	19	193	14	33	542
235-10	2	5	31	55	17	12	3	4	34	6	2	517
	2	4	39	48	11	3	3	9	37	0	3	511
231-10	0	12	51	33	47	32	10	13	21	11	33	497
	0	14	66	54	31	42	10	10	22	11	22	470
240-10	3	29	83	160	17	5	37	7	78	9	1	507
	3	23	59	163	11	3	28	8	111	17	3	561
246-10	1	29	152	84	14	11	12	37	50	18	6	585
	2	28	136	70	12	14	23	19	52	16	5	589

Sham

Median

Animal	R10	SIVmac239 Gag	SIVmac239 Env1	SIVmac239 Env2	SIVmac239 Pol1	SIVmac239 Pol2	SIVsm543 Gag	SIVsm543 Env1	SIVsm543 Env2	SIVsm543 Pol1	SIVsm543 Pol2	PHAM
217-10	1	1	1	2	6	2	0	2	1	0	2	558
224-10	3	1	1	3	5	5	1	1	1	0	1	518
228-10	1	2	0	0	1	2	1	0	0	1	0	504
230-10	2	1	5	3	2	1	1	2	0	1	1	541
236-10	1	2	0	1	0	1	0	0	0	0	0	528
237-10	1	1	0	1	5	1	0	2	1	2	1	557
238-10	1	1	0	1	1	1	1	0	0	1	1	550
241-10	0	0	0	1	3	2	1	0	2	0	0	527
242-10	0	0	1	0	1	0	0	0	0	0	0	340
243-10	1	0	0	0	1	1	1	0	1	0	1	413

Data

Animal	R10	SIVmac239 Gag	SIVmac239 Env1	SIVmac239 Env2	SIVmac239 Pol1	SIVmac239 Pol2	SIVsm543 Gag	SIVmac239 Env1	SIVsm543 Env2	SIVsm543 Pol1	SIVsm543 Pol2	PHAM
217-10	0	1	1	0	3	4	0	0	1	0	3	552
	2	1	0	3	8	0	0	3	0	0	1	563
224-10	3	0	2	0	6	5	1	1	1	0	0	517
	2	1	0	5	4	4	0	1	1	0	1	518
228-10	1	1	0	0	1	1	1	0	0	1	0	495
	0	2	0	0	0	2	0	0	0	0	0	513
230-10	2	2	5	2	2	1	2	3	0	1	1	549
	2	0	5	4	2	1	0	0	0	0	0	532
236-10	1	1	0	0	0	0	0	0	0	0	0	520
	0	3	0	1	0	1	0	0	0	0	0	535
237-10	1	1	0	1	2	1	0	2	0	0	1	577
	0	0	0	1	8	1	0	1	1	3	0	537
238-10	1	1	0	0	1	2	0	0	0	1	1	528
	1	0	0	1	1	0	2	0	0	1	0	572
241-10	0	0	0	2	2	1	1	0	3	0	0	572
	0	0	0	0	3	2	0	0	0	0	0	482
242-10	0	0	1	0	0	0	0	0	0	0	0	331
	0	0	0	0	1	0	0	0	0	0	0	348
243-10	1	0	0	0	1	1	0	0	0	0	0	422
	0	0	0	0	0	0	1	0	1	0	1	403

Supplementary Table 2. P values Following Fisher's Exact/Permutation Tests of All Informative Sites

Amino Acid Site	Sham vs. Stock	Ad35/Ad26 vs. Stock	Ad35/Ad26/Env vs. Stock	Ad35/Ad26 vs. Sham	Ad35/Ad26/Env vs. Sham	Ad35/Ad26 vs. Ad35/Ad26/Env	Ad35/Ad26 + Ad35/Ad26/Env vs. Stock	Ad35/Ad26 + Ad35/Ad26/Env vs. Sham
23	0.016	0.007	0.018	0.392	0.390	1.000	**0.001**	0.258
45	0.013	**<0.001**	**0.002**	0.032	0.090	1.000	**<0.001**	0.013
47	0.181	**<0.001**	**<0.001**	**0.001**	0.007	1.000	**<0.001**	**<0.001**
70	**<0.001**	**<0.001**	**<0.001**	**0.001**	0.008	1.000	**<0.001**	**<0.001**
130	0.670	0.653	1.000	0.582	1.000	1.000	1.000	0.642
132	1.000	0.768	0.449	0.771	0.451	1.000	0.427	0.433
133	0.574	0.345	0.170	0.650	0.103	0.070	1.000	0.679
134	0.831	0.569	0.448	0.773	0.448	1.000	0.297	0.432
137	0.670	0.380	1.000	0.159	1.000	0.541	0.688	0.377
138	0.132	0.160	0.198	1.000	0.034	0.040	0.603	0.371
139	1.000	0.784	0.448	0.787	0.286	0.647	0.447	0.331
140	0.481	0.652	0.055	1.000	0.015	0.035	0.680	0.237
141	0.481	0.652	0.055	1.000	0.015	0.035	0.680	0.237
142	0.595	0.652	0.055	1.000	0.016	0.035	0.680	0.322
143	0.598	0.821	0.032	1.000	0.015	0.035	0.540	0.237
144	0.821	1.000	0.679	0.771	0.451	1.000	0.583	0.433
154	0.118	0.539	0.338	0.672	1.000	1.000	0.567	0.480
161	0.038	0.784	0.105	0.060	0.776	0.137	0.644	0.222
185	0.841	0.464	1.000	0.326	0.740	1.000	0.519	0.387
186	**0.005**	0.539	0.019	0.102	0.702	0.095	0.157	0.399
208	1.000	1.000	1.000	1.000	1.000	1.000	1.000	1.000
355	0.479	0.661	0.578	1.000	1.000	1.000	0.398	1.000
380	0.306	0.436	1.000	1.000	0.480	0.445	0.501	1.000
424	0.271	0.613	0.723	0.164	1.000	0.460	1.000	0.318
428	0.284	0.330	0.726	0.064	1.000	0.285	0.823	0.161
433	0.644	0.763	0.679	1.000	0.451	0.647	1.000	0.794
486	0.211	1.000	0.019	0.319	0.136	0.030	0.304	1.000
721	0.592	1.000	0.356	1.000	0.427	0.375	0.546	1.000
726	0.497	0.320	1.000	1.000	1.000	1.000	0.411	1.000
760	1.000	1.000	0.338	1.000	0.437	0.326	1.000	1.000
762	0.606	1.000	0.042	1.000	0.015	0.030	0.400	0.298
763	0.536	0.303	0.035	0.099	0.091	0.008	0.715	1.000
770	1.000	0.512	0.382	0.519	0.386	0.128	1.000	1.000
780	0.621	0.539	1.000	1.000	1.000	1.000	1.000	1.000
805	0.494	0.391	0.019	0.830	0.044	0.099	0.079	0.246
808	0.490	0.375	0.038	0.660	0.080	0.285	0.073	0.232
820	0.864	0.271	0.250	0.382	0.386	1.000	0.120	0.181
830	1.000	0.549	1.000	0.552	1.000	1.000	0.267	0.280
881	0.233	0.813	1.000	0.601	0.502	1.000	0.832	0.495
885	0.540	1.000	1.000	0.601	0.502	1.000	0.828	0.495
888	0.160	0.813	0.759	0.427	0.290	0.730	1.000	0.255

Note: After Bonferroni corrections, p-values<0.00625 considered significant
